# Identify differential gene expressions in fatty infiltration process in rotator cuff

**DOI:** 10.1186/s13018-019-1182-1

**Published:** 2019-05-28

**Authors:** Pengfei Hu, Lifeng Jiang, Lidong Wu

**Affiliations:** 1 Department of Orthopedic Surgery, Second Affiliated Hospital, School of Medicine, Zhejiang University, 88 Jiefang Road, Hangzhou, Zhejiang 310009 People’s Republic of China; 2 Orthopedics Research Institute of Zhejiang University, Hangzhou, People’s Republic of China

**Keywords:** Rotator cuff tears, Fatty infiltration, DEGs, GO annotation, KEGG pathway, Module analysis

## Abstract

**Background:**

Rotator cuff tears are one of the most frequent upper extremity injuries and lead to pain and disability. Recent studies have implicated fatty infiltration in rotator cuff is a key failure element with the higher re-tear rates and poorer functional prognosis. Therefore, we investigated the differential expression of key genes in each stage of rotator cuff tear.

**Methods:**

A published expression profile was downloaded from the Gene Expression Omnibus database and analyzed using the Linear Models for Microarray Data (LIMMA) package in R language to identify differentially expressed genes (DEGs) in different stages of injured rotator cuff muscles. Gene ontology (GO) functional and Kyoto Encyclopedia of Genes and Genomes (KEGG) pathway analyses were performed to annotate the function of the DEGs. Finally, PPI network and module analysis were used to identify hub genes.

**Results:**

A total of 1089 fatty infiltration-related DEGs were identified, including 733 upregulated and 356 downregulated genes, and GO analyses confirmed that fatty infiltration was strongly associated with inflammatory response, aging, response to lipopolysaccharide, and immune response. Significantly enriched KEGG pathways associated with these DEGs included the phagosome, cell adhesion molecules, tuberculosis, and osteoclast differentiation. Further analyses via a PPI network and module analysis identified a total of 259 hub genes. Among these, Tmprss11d, Ptprc, Itgam, Mmp9, Tlr2, Il1b, Il18, Ccl5, Cxcl10, and Ccr7 were the top ten hub genes.

**Conclusions:**

Our findings indicated the potential key genes and pathways involved in fatty degeneration in the development of fatty infiltration and supplied underlying therapeutic targets in the future.

**Electronic supplementary material:**

The online version of this article (10.1186/s13018-019-1182-1) contains supplementary material, which is available to authorized users.

## Background

Rotator cuff tear (RCT) is one of the most common musculoskeletal disorder in orthopedics department. Earlier in 1991, the prevalence of shoulder disorders in people aged 70 and above was 21% [1]. According to a survey performed by Hiroshi in 2012, the prevalence of RCT was 22.1% was in the general population, and the prevalence was significantly increased with advanced age [2]. The disease is characterized as injury of one or more tendons/muscles in rotator cuff, which can cause pain, stiffness, muscle weakness, and functional limitation. Common risk factors for RCT include advancing age, overuse of shoulder, trauma, and shoulder arthritis [3].

Surgery is an effective solution for patients with large and full-thickness RCT. Currently, surgical procedures include mini-open and all-arthroscopic rotator cuff repair. According to the follow-up results, there is no significant difference in clinical prognosis between the two methods [4]. However, the failure rate was reported ranged from 23 to 77% in arthroscopically repaired massive rotator cuff tears, which characterized as the size of tear was more than 5 cm or the tear involving more than two cuff tendons [5–7]. The size of the defect, tendon retraction, muscular atrophy, and degree of fatty degeneration were regarded as failure elements and resulted in higher rate of no-heal and re-tear [8, 9]. Fatty infiltration was introduced and staged by Goutallier et al. in 1994 and was viewed as a key factor responsible for the higher re-tear rates and poorer functional prognosis [10, 11]. Fatty degeneration of the infraspinatus was reported as the most independent predictor on outcomes following rotator cuff repair [12]. Chung et al. also found fatty infiltration of the infraspinatus is a significantly positive correlated factor with healing failure rate after arthroscopic repair of a massive rotator cuff tear [6]. Another cohort study performed by Gladstone et al. demonstrated that both muscle atrophy and fatty infiltration of the rotator cuff muscles cast significant impacts on regulating cuff repair and functional recovery [13]. Therefore, an increasing large number of research groups are focusing on the specific mechanism of fatty infiltration.

Till now, the key genes and pathway involved in the fatty infiltration of rotator cuff tear still unclear. Ren et al. identified differentially expressed genes and signaling pathways involved in rotator cuff tear through bioinformatics analysis [14]. Their study focused on the different genes between torn tendon samples and intact samples. In the present study, to explore differential gene expression of the differential stage of RCT, we examined one published gene dataset available in the Gene Expression Omnibus (GEO) database. Differential expression of genes during each developmental stages of massive rotator cuff tear was examined by comprehensive bioinformatics analyses. Then Gene Ontology (GO) and Kyoto Encyclopedia of Genes and Genomes (KEGG) pathway enrichment analyses were performed, and a protein–protein interaction (PPI) network was used to screen for crucial genes and pathways in the pathophysiological processes of fatty infiltration. Chief sub-PPI networks are explored by MCODE.

## Methods

### Expression profile dataset

Gene expression profiles for rats with a massive tear of the rotator cuff at different development stages (GSE103266) was obtained from the GEO database. In this experiment, rats were established by a bilateral full-thickness supraspinatus tear and suprascapular neurectomy and divided into four groups according to the period after operation: 0 days (un-operated controls), 10 days, 30 days, and 60 days post-injury. The count data in GSE103266 was normalized by DESeq2 package in R software and presented in log_2_(TPM) [15]. The quality of gene expression data was analyzed and visualized using the ggplot2 package of R software for each sample. This research has been approved by the IRB of the authors’ affiliated institutions.

### Differential gene expression analyses

We compared the different three stages of rats (10 days, 30 days, and 60 days after operation) with un-operated controls to identify the DEGs. The Linear Models for Microarray Data (LIMMA) (limma) package, which includes lmFit, eBayes, and topTable functions, was used for pairwise comparison of DEGs [16]. *P* < 0.05 and abs(log_2_) fold change (FC) > 1 were used as the cutoff criteria.

### GO annotation and KEGG pathway enrichment analyses of DEGs

GO annotation is a classic method used in bioinformatics analyses to describe the normal, in vivo biological role of genes or gene products [17]. KEGG, a collection of databases representing our knowledge on the molecular interaction, reaction, and relation networks, was used to clarify the potential role of the DEGs [18]. GO functional analyses encompassing biological processes (BP), cell components (CC), and molecular functions (MF) was performed using the Database for Annotation, Visualization and Integrated Discovery (DAVID ver. 6.8), with *P* < 0.05 used as a cutoff.

### PPI network construction and hub gene identification

A PPI network for the DEGs identified above was built based on The Search Tool for the Retrieval of Interacting Genes (STRING, http://string-db.org) and Cytoscape (ver. 3.5.1) [19]. In the PPI network, genes served as “nodes” and the line segment between two nodes represented associated interactions. The color of the lines between genes indicates the degree of interaction. The Centiscape plugin was used to determine the degree of connectivity for each node in the PPI network. Genes with a degree >10 were defined as hub genes. Furthermore, module analysis was performed by using the MCODE plugin (version 1.3) to explore the most important clustering modules in the huge PPI network (degree cutoff = 5, k-core = 2, node score cutoff = 0.2, and max. Depth rom Seed: 100).

## Results

### Data distribution analyses and DEG screening

**Fig. 1:**
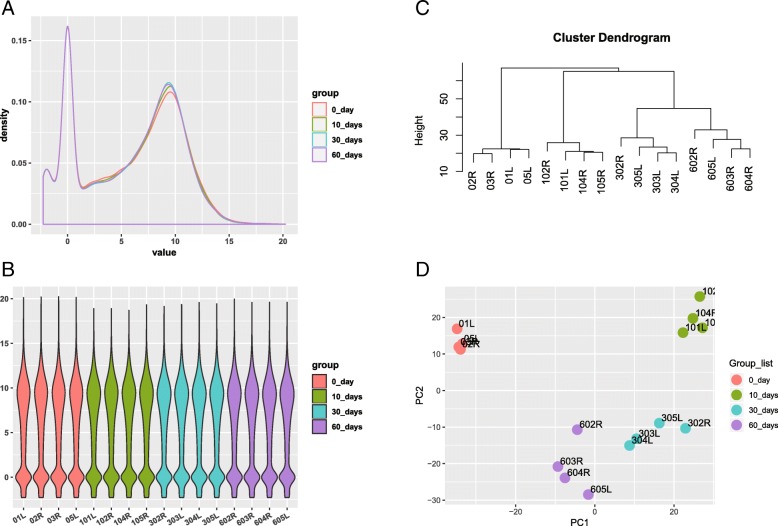
Distribution analyses of gene expression. **a** Distribution of gene expression levels in GSE103266. **b** Distribution of genes expression of 16 samples. **c** Cluster analyses for all samples. **d** Principle component analyses of each samples

In GSE103266, a total of 17147 genes were detected in 16 samples. The expression values (ranging from 0 to 20 log_2_ (TPM)) and the distributions were similar between the four groups (Fig. 1a, b). H-cluster analyses and principal component showed that samples were easily grouped into different groups (Fig. 1c, d). Confirmed by these data distribution analyses, further bioinformatics analyses could be performed based on the available data.

**Fig. 2:**
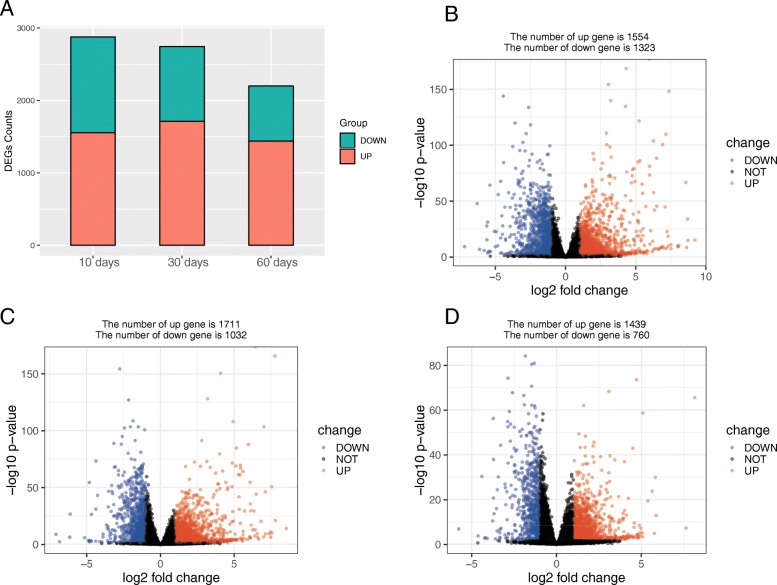
**a** Overview of DEGs in three comparisons. **b** Volcano plots displaying of group 0 day vs. group 10 days. **c** Volcano plots displaying of group 0 day vs. group 30 days. **d** Volcano plots displaying of group 0 day vs. group 60 days

Following data processing using limma, we identified 2877 DEGs (1554 upregulated, 1323 downregulated) in group 0 day vs. group 10 days (Fig. 2b), 2743 DEGs (1711 upregulated, 1032 downregulated) in group 0 day vs. group 30 days (Fig. 2c), and 2199 DEGs (1439 upregulated, 760 downregulated) in group 0 day vs. group 60 days (Fig. 2d).

### Intersection between the DEGs

**Fig. 3:**
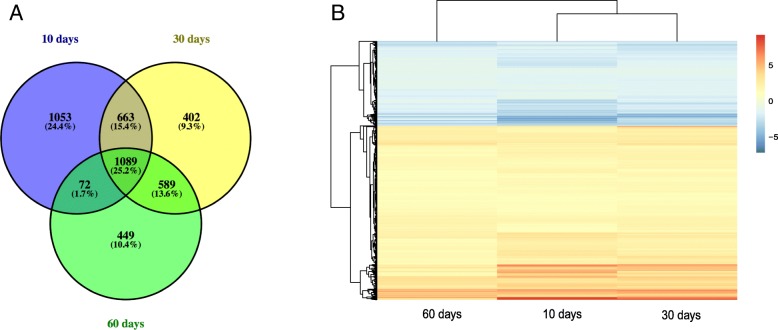
**a** Venn diagram of DEGs across different stages. **b** Hierarchical clustering analyses of DEGs common to all groups

We identified a total of 1089 DEGs common across each of the different stages by intersecting the three comparisons above (Fig. 3a), including 733 upregulated and 356 downregulated genes (Additional file 1: Table S1). Hierarchical clustering of the identified DEGs is displayed as a heatmap in Fig. 3b.

### GO annotation and KEGG pathway enrichment analyses

**Fig. 4:**
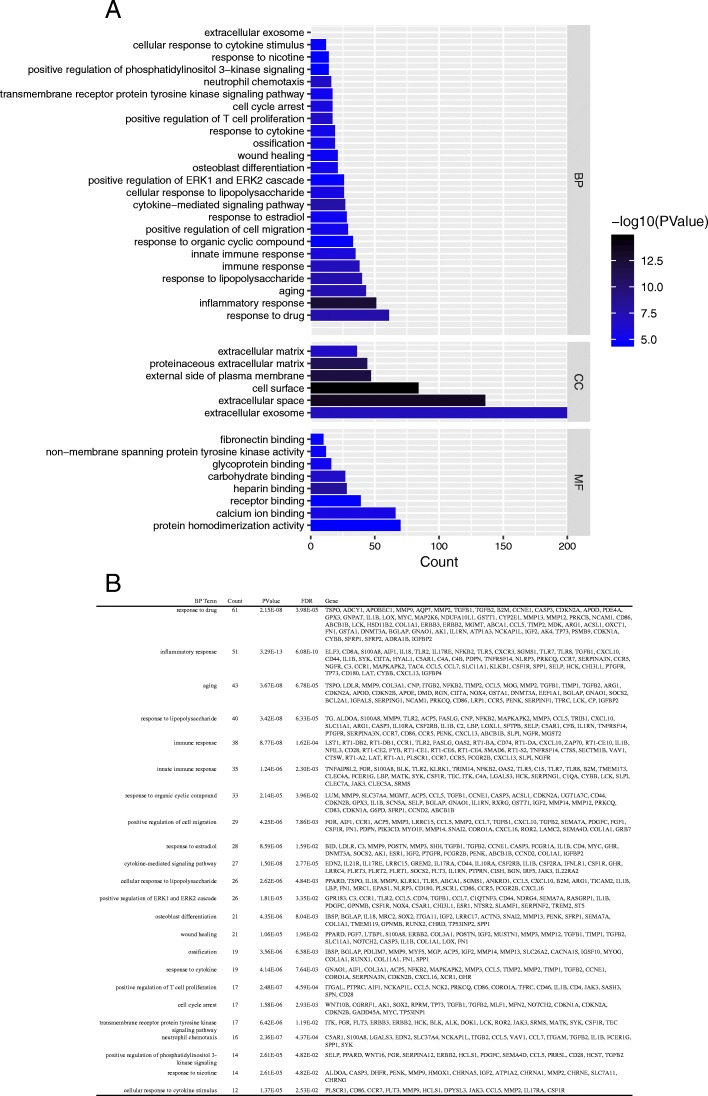
**a** Histogram of Gene Ontology (GO) functional classification of DEGs. GO terms including biological processes (BP), cellular components (CC), and molecular functions (MF). **b** Top BP terms and their corresponding genes in GO functional enrichment analyses

**Fig. 5:**
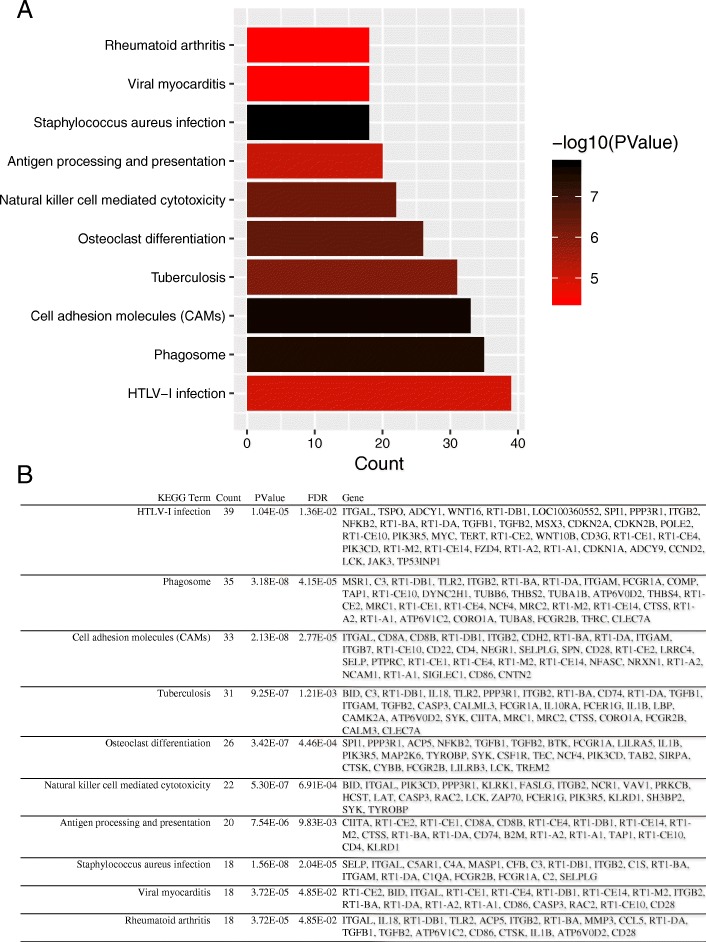
**a** Histogram of KEGG pathways enrichment in DEGs. The graph displays only significantly enriched KEGG terms (*P* < 0.05), with darker red indicating greater significance. **b** Individual KEGG terms and their corresponding genes are shown for each group

GO annotation and KEGG pathway enrichment analyses were performed to better understand the functional significance of the DEGs. DEGs mainly partcipated in biological processes (BP) regarding response to drug, inflammatory response, aging, response to lipopolysaccharide and immune response. Significant cellular component (CC) terms revealed DEGs were principally enriched in extracellular exosome, extracellular space, and cell surface. Finally, the top five molecular functions (MF) identified were protein homodimerization activity, calcium ion binding, receptor binding, heparin binding, and carbohydrate binding (Fig. 4a). All significant biological processes-enriched entries are shown in a histogram in Fig. 4b. KEGG analyses of the DEGs revealed mainly enrichment for ten terms (Fig. 5a). The top five significantly enriched KEGG pathways were the HTLV-I infection, phagosome, cell adhesion molecules, tuberculosis, and osteoclast differentiation. Detailed information regarding each of the DEGs involved in the KEGG pathway are listed in Fig. 5b.

**Fig. 6:**
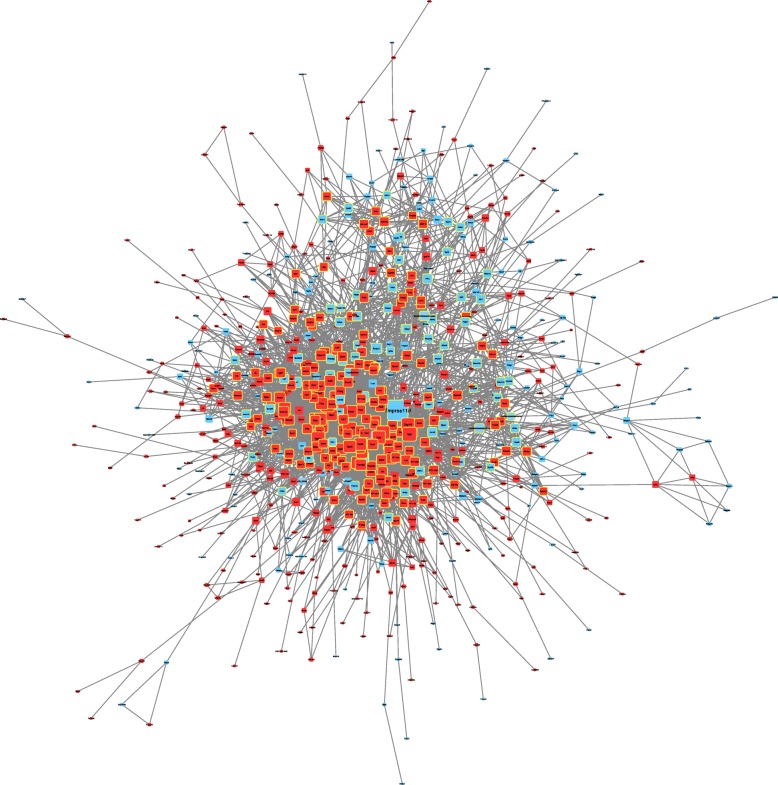
Protein–protein interaction (PPI) network construction. Genes with yellow circles represent hub genes with a degree of interaction ≥ 10. A total of 259 DEGs were identified as fatty infiltration-related hub genes

### PPI network construction and module analysis

We constructed a putative PPI network map for the overlapping DEGs using the STRING database and Cytoscape(Fig. 6). After degree calculation, a total of 259 hub genes were identified with the degree more than 10, as listed in Additional file 2: Table S2. Among these, Tmprss11d, Ptprc, Itgam, Mmp9, Tlr2, Il1b, Il18, Ccl5, Cxcl10, and Ccr7 were the top ten hub genes according to the MCODE degree score.

**Fig. 7:**
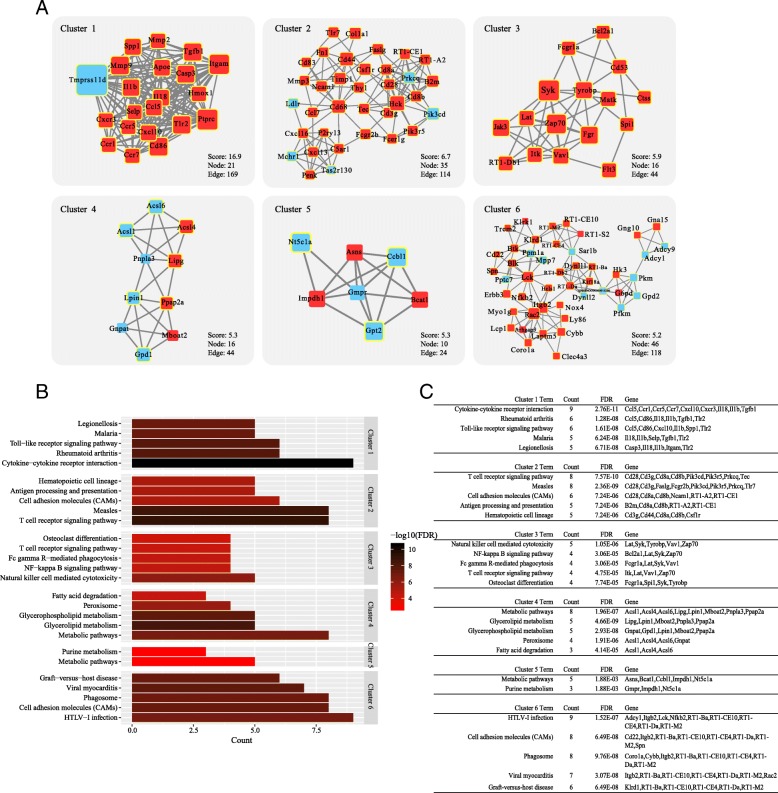
**a** The significant six modules in the PPI network with both MCODE score ≥ 5 and nodes ≥ 5. **b** Histogram of KEGG pathways enrichment for these six modules. **c** Individual KEGG terms and their corresponding genes

A total of six modules were separated from the PPI network with both MCODE score ≥ 5 and nodes ≥ 5 (Fig. 7). KEGG analysis showed that the main pathways of these modules were associated with cytokine-cytokine receptor interaction, rheumatoid arthritis and toll-like receptor signaling pathway, osteoclast differentiation, metabolic pathways, fatty acid degradation, and so on.

## Discussion

The mechanism of fatty infiltration of the rotator cuff is complex and still unclear. Using a mouse model constructed by unilateral supraspinatus and infraspinatus tendon tear, Wang et al. demonstrated atrophy and fatty infiltration of muscle exist in the 6-week delayed repair group. And the severity of fatty infiltration was closely linked with the shoulder function [20]. Another massive rotator cuff tear model caused by a full-thickness tenectomy of the right supraspinatus and infraspinatus in rats showed that the muscle fiber force is decreased and expressions of fibrosis and lipid accumulation related genes were increased [21]. A further study performed by Liu et al. demonstrated that the main cellular sources involved in fatty infiltration were Tie2 + progenitor cells and fibro/adipogenic progenitor cells [22]. These three studies were consistent with the clinical reports and researches. In a prospective study aimed to massive rotator cuff tears patients younger than 65 years, 67.9% were demonstrated with a stage 3 or 4 fatty infiltration of the supraspinatus muscle and 47.3% were suffered from a stage 3 or 4 fatty infiltration of the infraspinatus muscle [23]. And patients with a high degree of fatty infiltration exhibited poorer prognosis after surgery. Patients with grade 3 fatty degeneration showed significant improvement after arthroscopic rotator cuff repair compared to those patients who graded as Goutallier stage IV after nearly 3 years of follow-up [24]. However, a successful repair of rotator cuff would not bring a significant improvement of fatty infiltration and atrophy of the rotator cuff [13]. So, studies focused on the cellular and molecular basis of fatty infiltration are of great significance in the prevention and treatment of fatty infiltration in rotator cuff tear.

In the present study, a bioinformatics analysis was used to analyze an available database downloaded from GEO. According to our results, a total of 1089 DEGs were identified as the key mediators in the pathophysiological processes of fatty infiltration. Moreover, we also performed GO annotation and KEGG pathway enrichment analyses to potential biological functions and pathways involved in fatty infiltration. In addition, 259 hub genes including Tmprss11d, Ptprc, Itgam, Mmp9, Tlr2, Il1b, Il18, Ccl5, Cxcl10, and Ccr7 and six modules with important functions were identified from the PPI network.

At present, there are only a few theories and research results about the mechanism of fatty infiltration in rotator cuff. Tmprss11d, a member of type II transmembrane serine protease (TTSP) family, was highly expressed in respiratory epithelium and proved to exert an important role in the host defense system [25]. Till now, the role of TMPRSS11D in the musculoskeletal system was not clear. Cao et al. reported higher expression of TMPRSS11D is closely linked with the severity of the cancer because of its ability to disintegrate the extracellular matrix [26]. TMPRSS11D was also demonstrated to have a relationship with fat metabolism in the liver [25]. Therefore, it is an important research direction of the role of TMPRSS11D in the degeneration of tendon and lipid metabolism in the future. MMP family was also identified as a major player in rotator cuff tear. Protein expressions of MMP-9 and MMP-13 were notably increased in rotator cuff specimens obtained from surgery patients [27]. However, in MMP-13 (−/−) knockout mice group, researchers found a higher amount of fat infiltration compared to the wildtype MMP-13 (+/+) mice group. These interesting results indicated that MMP-13 exert a significant role in rotator muscle fatty infiltration and may be a potential target for treatment [28]. MMP inhibitor was helpful in the improvement of tendon-to-bone healing in a rotator cuff repair model [29]. Moreover, Tlr2, another identified hub gene in our study, was highly expressed in rotator cuff tendon in the first 2 weeks following injuries [30]. Tlr2 was viewed as an important mediator in NLRP3 inflammasome. The lowered activity of NLRP3 leads to a decrease in the fat accumulation in hepatocytes [31]. So, the molecular mediators associated with NLRP3 inflammasome was worth studying in the pathology of fatty infiltration in rotator cuff tendons. Taken together, hub genes identified in our research by comprehensive bioinformatic analyses were meaningful and worthy of deep-going study in the future.

We also performed an intensive module analysis based on the PPI network in this study. In chronically rotator cuff muscles, increased gene expressions of PPAR-γ, PLD1, miR-27a and lipid synthesis were obversed. And ECM degradation- and remodeling-related genes such as MMP2, MMP9, and MMP14 were also overexpressed in the pathological process of fibrosis [32]. Blocking of p38 MAPK signaling, an important regulator of adipogenesis, reduced the fat accumulation by nearly half and adjusted the expressions of adipogenesis- and matrix accumulation-related genes [33]. Our KEGG pathway enrichment analyses showed that the functions of module 4 and 5 were mainly associated with metabolic pathways and fatty acid degradation. And DEGs such as Acsl1, Acsl4, Acsl6, Lipg, Lpin1, Mboat2, Pnpla3, Ppap2a, Ccbl1, Impdh1, and Nt5c1a were functionality associated with these pathways.

A limitation of this study is that the stage of fatty infiltration in the rat models in GSE103266 is unclear. Besides, there are some anatomical differences between human and rat. Therefore, the pathophysiological process of fatty infiltration in rat is different from human. We still need to conduct validation experiment to proof our speculation in the future.

## Conclusion

The current study identified a series of DEGs in each stage of massive rotator cuff tear. Using a series of bioinformatics analyses, hub genes including Tmprss11d, Ptprc, Itgam, Mmp9, Tlr2, Il1b, Il18, Ccl5, Cxcl10, and Ccr7 were identified. Further GO and KEGG pathway analysis suggests potential mechanisms through which these hub genes mediate fatty infiltration pathogenesis in rotator cuff tear. In addition, module analysis highlights the important roles of metabolic pathways and fatty acid degradation in the regulation of fatty infiltration. Our results indicated the potential key genes and pathways involved in fatty degeneration and supplied therapeutic targets to improve clinical outcomes for patients who suffer from chronic rotator cuff tears and fatty infiltration.

## Additional files

Additional file 1: **Table S1**. 1089 DEGs in each of the different stages of rotator cuff tear. (DOCX 148 kb)

Additional file 2: **Table S2**. Hub-genes identified by CentiScape (degree ≥ 5) among 1089 DEGs. (DOCX 35 kb)

## Abbreviations

BP: Biological processes
CC: Cell components
DEGs: Differentially expressed genes
GEO: Gene Expression Omnibus
GO: Gene ontology
KEGG: Kyoto Encyclopedia of Genes and Genomes
LIMMA: Linear Models for Microarray data
MF: Molecular functions
PPI: Protein–protein interaction
RCT: Rotator cuff tear
