# Supplementary material for: Identify differential gene expressions in fatty infiltration process in rotator cuff
Source: J Orthop Surg Res. 2019 May 28;14:158. doi: 10.1186/s13018-019-1182-1 (PMC6537194; doi:10.1186/s13018-019-1182-1)
Supplement: Supplementary file 1 — Table S1. 1089 DEGs in each of the different stages of rotator cuff tear. (DOCX 148 kb) [file 13018_2019_1182_MOESM1_ESM.docx]

Table S1 1089 DEGs in each of the different stages of rotator cuff tear

| Gene symbol | 10_days | 30_days | 60_days | Change |
| --- | --- | --- | --- | --- |
| Odf3l2 | -7.20 | -7.08 | -5.76 | DOWN |
| Aqp4 | -6.30 | -3.14 | -2.04 | DOWN |
| Edn2 | -6.13 | -3.07 | -2.84 | DOWN |
| Klkb1 | -5.80 | -5.13 | -2.23 | DOWN |
| Ubxn10 | -5.62 | -6.12 | -3.54 | DOWN |
| Msx3 | -5.60 | -3.33 | -3.77 | DOWN |
| Iqcg | -5.56 | -2.26 | -1.40 | DOWN |
| Ropn1 | -5.43 | -3.57 | -2.99 | DOWN |
| Acsm5 | -5.39 | -2.87 | -1.85 | DOWN |
| Sftpb | -5.35 | -1.96 | -3.34 | DOWN |
| Slc16a3 | -5.08 | -3.41 | -2.45 | DOWN |
| Cish | -4.84 | -3.38 | -3.51 | DOWN |
| Dhrs7c | -4.81 | -2.06 | -1.44 | DOWN |
| Cpne6 | -4.63 | -3.15 | -4.63 | DOWN |
| Mlf1 | -4.63 | -2.90 | -2.20 | DOWN |
| Sec14l5 | -4.63 | -2.70 | -1.16 | DOWN |
| Asb18 | -4.54 | -2.29 | -1.62 | DOWN |
| Fscn2 | -4.51 | -3.65 | -2.12 | DOWN |
| Slc26a9 | -4.48 | -4.67 | -3.72 | DOWN |
| Gpd1 | -4.44 | -3.06 | -2.35 | DOWN |
| Mybpc2 | -4.42 | -2.65 | -1.57 | DOWN |
| Lmod1 | -4.33 | -4.27 | -2.61 | DOWN |
| Igfals | -4.28 | -3.60 | -2.46 | DOWN |
| Lgi2 | -4.27 | -3.08 | -2.11 | DOWN |
| Bai2 | -4.21 | -4.79 | -4.24 | DOWN |
| Shisa8 | -4.09 | -4.69 | -4.09 | DOWN |
| Mss51 | -4.06 | -3.16 | -2.26 | DOWN |
| Hapln2 | -4.02 | -2.48 | -2.33 | DOWN |
| Pfkfb1 | -3.96 | -2.36 | -1.14 | DOWN |
| Pde4a | -3.79 | -2.62 | -1.90 | DOWN |
| Cort | -3.77 | -2.74 | -1.82 | DOWN |
| Mylk2 | -3.76 | -3.12 | -1.96 | DOWN |
| Neu2 | -3.75 | -2.04 | -1.44 | DOWN |
| Adcy1 | -3.68 | -3.88 | -3.27 | DOWN |
| Fanca | -3.67 | -3.29 | -3.30 | DOWN |
| Cib2 | -3.55 | -2.60 | -1.70 | DOWN |
| Slc41a3 | -3.55 | -2.62 | -1.73 | DOWN |
| Jph2 | -3.50 | -3.34 | -2.74 | DOWN |
| Sbk2 | -3.49 | -3.50 | -1.39 | DOWN |
| Shh | -3.49 | -3.39 | -3.47 | DOWN |
| Esr1 | -3.49 | -2.00 | -2.24 | DOWN |
| Myo3b | -3.47 | -2.53 | -1.62 | DOWN |
| Espl1 | -3.39 | -2.79 | -1.95 | DOWN |
| St8sia5 | -3.38 | -2.85 | -2.73 | DOWN |
| Pla2g2c | -3.37 | -2.46 | -1.46 | DOWN |
| Nrep | -3.35 | -2.74 | -2.69 | DOWN |
| Syndig1l | -3.33 | -3.12 | -3.34 | DOWN |
| Masp1 | -3.31 | -2.54 | -1.95 | DOWN |
| RGD1307461 | -3.30 | -1.79 | -1.73 | DOWN |
| Kcna10 | -3.28 | -4.89 | -2.60 | DOWN |
| Sema7a | -3.27 | -3.24 | -2.85 | DOWN |
| Tpx2 | -3.26 | -3.16 | -2.86 | DOWN |
| Myoz1 | -3.25 | -1.85 | -1.06 | DOWN |
| Exoc3l4 | -3.24 | -1.69 | -1.26 | DOWN |
| Slc38a5 | -3.24 | -1.85 | -2.51 | DOWN |
| Cmbl | -3.24 | -1.84 | -1.08 | DOWN |
| Ak1 | -3.23 | -1.21 | -1.12 | DOWN |
| Actn3 | -3.21 | -2.25 | -1.90 | DOWN |
| Unc5a | -3.19 | -4.37 | -4.40 | DOWN |
| Rims4 | -3.19 | -4.79 | -4.19 | DOWN |
| Car12 | -3.18 | 2.05 | 3.64 | DOWN |
| Kcna7 | -3.17 | -2.41 | -1.13 | DOWN |
| RGD1565323 | -3.15 | -4.82 | -3.44 | DOWN |
| Gnao1 | -3.14 | -2.84 | -2.02 | DOWN |
| Dusp8 | -3.09 | -2.58 | -1.16 | DOWN |
| Ankrd9 | -3.06 | -2.14 | -1.88 | DOWN |
| Acsl6 | -3.05 | -1.84 | -1.62 | DOWN |
| Lingo4 | -3.04 | -3.52 | -2.00 | DOWN |
| Ush1c | -3.02 | -1.26 | -1.48 | DOWN |
| Nfasc | -3.01 | -2.59 | -1.93 | DOWN |
| Ldhd | -3.00 | -1.57 | -1.13 | DOWN |
| Rap1gap2 | -2.95 | -2.39 | -1.94 | DOWN |
| Grtp1 | -2.94 | -1.68 | -1.11 | DOWN |
| Map2k6 | -2.92 | -1.53 | -1.24 | DOWN |
| Arrdc2 | -2.89 | -2.50 | -1.03 | DOWN |
| Afap1l1 | -2.89 | -1.93 | -1.45 | DOWN |
| Rasgrp3 | -2.87 | -1.70 | -1.14 | DOWN |
| Pnpla3 | -2.85 | -1.83 | -1.19 | DOWN |
| Dagla | -2.81 | -2.59 | -1.78 | DOWN |
| LOC691083 | -2.80 | -1.65 | -1.54 | DOWN |
| Tmem132b | -2.77 | -6.14 | -1.59 | DOWN |
| Pfkfb3 | -2.77 | -3.54 | -2.47 | DOWN |
| Asb10 | -2.76 | -2.14 | -1.47 | DOWN |
| Eepd1 | -2.75 | -2.76 | -2.39 | DOWN |
| Pfkm | -2.70 | -2.10 | -1.33 | DOWN |
| Aqp7 | -2.68 | -1.75 | -1.65 | DOWN |
| Snrpn | -2.67 | -3.25 | -2.92 | DOWN |
| Spata20 | -2.67 | -1.84 | -1.42 | DOWN |
| Pygm | -2.64 | -2.19 | -1.55 | DOWN |
| Smyd2 | -2.63 | -1.97 | -1.64 | DOWN |
| Atp2a1 | -2.62 | -2.18 | -1.73 | DOWN |
| Pik3c2g | -2.62 | -1.98 | -1.90 | DOWN |
| Pkm | -2.58 | -1.99 | -1.49 | DOWN |
| Dzip1l | -2.57 | -2.66 | -2.42 | DOWN |
| Lgalsl | -2.57 | -1.55 | -1.04 | DOWN |
| Hs3st1 | -2.56 | -2.02 | -2.02 | DOWN |
| Cacna1e | -2.56 | -1.97 | -1.65 | DOWN |
| Pkia | -2.56 | -2.37 | -2.13 | DOWN |
| Grem2 | -2.54 | -1.26 | -1.62 | DOWN |
| Mn1 | -2.54 | -3.16 | -2.22 | DOWN |
| Pwwp2b | -2.52 | -1.82 | -1.36 | DOWN |
| Sar1b | -2.52 | -2.41 | -1.28 | DOWN |
| Rps6ka2 | -2.50 | -2.24 | -1.75 | DOWN |
| Slc37a4 | -2.50 | -1.37 | -1.29 | DOWN |
| Nr4a3 | -2.49 | -3.06 | -1.97 | DOWN |
| Fsd2 | -2.49 | -1.16 | -1.01 | DOWN |
| Slamf1 | -2.49 | -1.75 | -2.12 | DOWN |
| Atp2b3 | -2.49 | -1.41 | -1.40 | DOWN |
| Gpcpd1 | -2.49 | -2.59 | -1.50 | DOWN |
| Ccdc28b | -2.49 | -1.63 | -1.08 | DOWN |
| Fzd4 | -2.48 | -2.02 | -1.44 | DOWN |
| Sgsm1 | -2.48 | -1.35 | -1.05 | DOWN |
| Rgn | -2.48 | -1.55 | -1.19 | DOWN |
| Tfrc | -2.48 | -1.36 | -1.15 | DOWN |
| Klhdc3 | -2.47 | -1.92 | -1.44 | DOWN |
| Hopx | -2.47 | -1.40 | -1.22 | DOWN |
| Slc25a25 | -2.45 | -3.03 | -1.77 | DOWN |
| Srcin1 | -2.43 | -3.04 | -3.69 | DOWN |
| Syt8 | -2.43 | -1.95 | -1.76 | DOWN |
| Paqr9 | -2.43 | -1.82 | -1.73 | DOWN |
| Grip2 | -2.42 | -1.24 | -1.03 | DOWN |
| Nefl | -2.41 | -4.38 | -2.65 | DOWN |
| Cacng7 | -2.41 | -4.37 | -3.73 | DOWN |
| Nt5c1a | -2.40 | -2.82 | -2.99 | DOWN |
| LOC500959 | -2.39 | -1.26 | -1.02 | DOWN |
| Atp1b2 | -2.38 | -2.57 | -2.88 | DOWN |
| Srrm3 | -2.36 | -2.07 | -1.21 | DOWN |
| Mstn | -2.35 | -2.95 | -3.50 | DOWN |
| Ace2 | -2.34 | -1.47 | -1.25 | DOWN |
| Car14 | -2.34 | -1.65 | -1.05 | DOWN |
| Wipf3 | -2.33 | -1.81 | -1.20 | DOWN |
| Srms | -2.33 | -1.66 | -1.22 | DOWN |
| Ppp1r1a | -2.30 | -1.90 | -1.32 | DOWN |
| Fam213b | -2.30 | -1.65 | -1.51 | DOWN |
| Tmem50b | -2.29 | -1.99 | -1.23 | DOWN |
| Lynx1 | -2.29 | -1.61 | -1.58 | DOWN |
| Ccbl1 | -2.29 | -1.41 | -1.08 | DOWN |
| Aldoa | -2.28 | -1.95 | -1.30 | DOWN |
| Fam160a1 | -2.27 | -1.65 | -1.14 | DOWN |
| Apba2 | -2.26 | -2.03 | -1.52 | DOWN |
| Inpp5j | -2.24 | -2.08 | -1.29 | DOWN |
| Wbscr17 | -2.24 | -1.52 | -1.31 | DOWN |
| Snurf | -2.23 | -1.40 | -1.20 | DOWN |
| Gucy2g | -2.20 | -1.98 | -1.01 | DOWN |
| Rxrg | -2.18 | -1.68 | -1.48 | DOWN |
| Kcnh2 | -2.16 | -1.08 | -1.05 | DOWN |
| Lpin1 | -2.14 | -2.84 | -2.78 | DOWN |
| Pdlim7 | -2.13 | -1.56 | -1.28 | DOWN |
| Susd4 | -2.13 | -1.82 | -2.30 | DOWN |
| Tmprss11d | -2.13 | -3.89 | -1.41 | DOWN |
| Gpd2 | -2.13 | -2.47 | -2.01 | DOWN |
| Slc25a34 | -2.11 | -1.62 | -1.57 | DOWN |
| Lgi1 | -2.11 | -2.52 | -1.12 | DOWN |
| Dnmt3a | -2.08 | -2.07 | -1.48 | DOWN |
| Kcnj12 | -2.07 | -1.45 | -1.51 | DOWN |
| Synpo2 | -2.06 | -1.84 | -1.26 | DOWN |
| Fbxl4 | -2.05 | -1.85 | -1.76 | DOWN |
| St3gal6 | -2.05 | -2.07 | -1.21 | DOWN |
| Eml1 | -2.04 | -2.24 | -1.89 | DOWN |
| Cpne4 | -2.03 | -2.43 | -1.67 | DOWN |
| Dusp26 | -2.02 | -2.75 | -1.65 | DOWN |
| Wnt16 | -2.02 | -2.65 | -1.87 | DOWN |
| Nfil3 | -2.02 | -1.39 | -1.16 | DOWN |
| Pdgfc | -2.00 | -1.24 | -1.68 | DOWN |
| Gpt2 | -2.00 | -2.29 | -2.60 | DOWN |
| Camk2a | -1.99 | -1.81 | -1.55 | DOWN |
| Mafb | -1.98 | -1.51 | -1.35 | DOWN |
| Arhgef37 | -1.98 | -1.68 | -1.18 | DOWN |
| Clic5 | -1.97 | -1.99 | -1.29 | DOWN |
| Magix | -1.96 | -1.86 | -1.57 | DOWN |
| Sfxn5 | -1.96 | -1.52 | -2.00 | DOWN |
| Phkg1 | -1.96 | -2.34 | -1.73 | DOWN |
| Stk32a | -1.94 | -2.70 | -2.18 | DOWN |
| Ntsr2 | -1.93 | -1.39 | -1.99 | DOWN |
| Agl | -1.92 | -1.13 | -1.00 | DOWN |
| Negr1 | -1.92 | -1.74 | -2.83 | DOWN |
| Agtpbp1 | -1.92 | -2.19 | -1.90 | DOWN |
| Prrg1 | -1.92 | -1.75 | -1.53 | DOWN |
| Trpm1 | -1.91 | -1.72 | -1.38 | DOWN |
| Mvb12b | -1.91 | -3.54 | -3.02 | DOWN |
| Myh4 | -1.90 | -1.95 | -2.48 | DOWN |
| Mapkapk2 | -1.89 | -1.93 | -1.39 | DOWN |
| Gmpr | -1.87 | -1.42 | -1.03 | DOWN |
| Plcd4 | -1.87 | -1.74 | -1.51 | DOWN |
| Mbnl1 | -1.85 | -1.90 | -1.34 | DOWN |
| Prkcq | -1.85 | -1.41 | -1.02 | DOWN |
| Mfsd6 | -1.84 | -1.59 | -1.05 | DOWN |
| Fam189b | -1.83 | -2.03 | -1.84 | DOWN |
| RGD1562658 | -1.82 | -1.59 | -1.40 | DOWN |
| Epas1 | -1.82 | -2.01 | -1.19 | DOWN |
| Adcy9 | -1.81 | -3.31 | -2.49 | DOWN |
| Asb4 | -1.81 | -1.85 | -1.51 | DOWN |
| Synpo | -1.79 | -2.62 | -1.41 | DOWN |
| RGD1309821 | -1.79 | -2.20 | -1.91 | DOWN |
| Arid3b | -1.78 | -1.91 | -1.08 | DOWN |
| Cdadc1 | -1.77 | -1.68 | -1.31 | DOWN |
| Slc9a2 | -1.77 | -1.62 | -1.63 | DOWN |
| Nmnat3 | -1.74 | -1.23 | -1.35 | DOWN |
| MGC116197 | -1.74 | -1.11 | -1.18 | DOWN |
| Ush1g | -1.74 | -1.40 | -2.04 | DOWN |
| Mchr1 | -1.73 | -1.23 | -1.72 | DOWN |
| RGD1311863 | -1.73 | -2.03 | -1.59 | DOWN |
| Klhl21 | -1.71 | -2.25 | -1.69 | DOWN |
| St3gal1 | -1.70 | -2.64 | -2.71 | DOWN |
| Pik3cd | -1.67 | -1.41 | -1.40 | DOWN |
| Prkg1 | -1.67 | -2.22 | -1.90 | DOWN |
| Mpp7 | -1.66 | -1.53 | -1.02 | DOWN |
| Lrrc20 | -1.64 | -2.14 | -1.47 | DOWN |
| Gdf1 | -1.64 | -1.31 | -1.38 | DOWN |
| Stradb | -1.64 | -1.53 | -1.23 | DOWN |
| Oxct1 | -1.64 | -1.53 | -1.18 | DOWN |
| Macrod1 | -1.63 | -1.19 | -1.02 | DOWN |
| Fgf1 | -1.62 | -1.91 | -1.45 | DOWN |
| RGD1562136 | -1.62 | -1.58 | -1.29 | DOWN |
| Trib1 | -1.62 | -1.45 | -1.43 | DOWN |
| Gnpat | -1.62 | -1.60 | -1.00 | DOWN |
| Ppapdc3 | -1.61 | -1.14 | -1.08 | DOWN |
| Asb2 | -1.60 | -2.44 | -1.75 | DOWN |
| Pdk2 | -1.59 | -1.58 | -1.33 | DOWN |
| Grhpr | -1.59 | -1.23 | -1.23 | DOWN |
| Ttc7b | -1.58 | -1.70 | -1.21 | DOWN |
| Llgl2 | -1.58 | -1.90 | -1.63 | DOWN |
| Jph1 | -1.58 | -2.15 | -1.86 | DOWN |
| Nhlrc1 | -1.57 | -2.04 | -1.83 | DOWN |
| Zfpm1 | -1.57 | -2.33 | -1.75 | DOWN |
| Tp53inp2 | -1.57 | -1.33 | -1.23 | DOWN |
| Ak4 | -1.57 | -2.41 | -2.62 | DOWN |
| Tmcc3 | -1.56 | -1.62 | -1.20 | DOWN |
| Scn4b | -1.56 | -1.99 | -1.25 | DOWN |
| Phospho1 | -1.56 | -2.39 | -1.46 | DOWN |
| RGD1561161 | -1.55 | -2.54 | -1.81 | DOWN |
| Mybpc3 | -1.55 | -1.53 | -3.34 | DOWN |
| Sobp | -1.55 | -2.37 | -2.50 | DOWN |
| Nptx1 | -1.55 | -2.32 | -1.37 | DOWN |
| Lrrc14b | -1.54 | -1.77 | -1.39 | DOWN |
| Prkaa2 | -1.54 | -2.00 | -1.72 | DOWN |
| Pcyox1 | -1.54 | -1.96 | -2.42 | DOWN |
| LOC100360552 | -1.54 | -1.74 | -1.32 | DOWN |
| Rnf150 | -1.53 | -1.55 | -1.70 | DOWN |
| Cryga | -1.51 | -1.06 | -1.14 | DOWN |
| Fkbp4 | -1.51 | -1.74 | -1.20 | DOWN |
| Satb1 | -1.50 | -1.56 | -1.09 | DOWN |
| Cited4 | -1.50 | -1.45 | -1.63 | DOWN |
| LOC294154 | -1.50 | -1.76 | -1.34 | DOWN |
| Ankrd34a | -1.50 | -1.51 | -1.49 | DOWN |
| Cdnf | -1.49 | -1.68 | -1.62 | DOWN |
| Calm3 | -1.48 | -1.86 | -2.02 | DOWN |
| Tmem38a | -1.47 | -1.32 | -1.22 | DOWN |
| Ccnd2 | -1.47 | -1.25 | -1.22 | DOWN |
| Plcl2 | -1.47 | -1.67 | -1.45 | DOWN |
| Tuba8 | -1.46 | -2.28 | -1.51 | DOWN |
| Dlat | -1.45 | -1.11 | -1.03 | DOWN |
| Pptc7 | -1.45 | -1.31 | -1.40 | DOWN |
| Slc25a30 | -1.45 | -1.86 | -1.82 | DOWN |
| Rassf3 | -1.44 | -1.28 | -1.02 | DOWN |
| RGD1306772 | -1.44 | -1.34 | -1.25 | DOWN |
| Hyal1 | -1.43 | -1.97 | -1.23 | DOWN |
| Mdga1 | -1.42 | -1.24 | -1.95 | DOWN |
| Ghr | -1.42 | -1.70 | -1.40 | DOWN |
| Zbtb7b | -1.42 | -2.42 | -1.93 | DOWN |
| Zfp91 | -1.42 | -1.72 | -1.28 | DOWN |
| Socs2 | -1.42 | -1.29 | -1.02 | DOWN |
| Sorl1 | -1.41 | -1.34 | -1.12 | DOWN |
| Ldlr | -1.41 | -1.45 | -1.60 | DOWN |
| Iqsec3 | -1.39 | -1.15 | -1.25 | DOWN |
| Rnf144b | -1.38 | -1.71 | -1.06 | DOWN |
| Wwp1 | -1.38 | -1.46 | -1.29 | DOWN |
| Gbas | -1.38 | -1.48 | -1.37 | DOWN |
| Eif4e3 | -1.37 | -1.61 | -1.62 | DOWN |
| Cacna1s | -1.36 | -1.61 | -1.15 | DOWN |
| Homer2 | -1.36 | -1.53 | -1.51 | DOWN |
| Ttc19 | -1.35 | -1.31 | -1.19 | DOWN |
| Hist2h2ab | -1.35 | -1.49 | -1.23 | DOWN |
| Slc25a23 | -1.35 | -1.47 | -1.55 | DOWN |
| Fam53a | -1.34 | -1.26 | -1.10 | DOWN |
| Tm2d2 | -1.33 | -1.13 | -1.11 | DOWN |
| Kif5a | -1.32 | -1.22 | -1.19 | DOWN |
| Cgrrf1 | -1.32 | -1.24 | -1.08 | DOWN |
| Narf | -1.31 | -1.41 | -1.01 | DOWN |
| Map3k10 | -1.31 | -1.65 | -1.74 | DOWN |
| Zfand3 | -1.31 | -1.59 | -1.37 | DOWN |
| RGD1306119 | -1.30 | -1.82 | -1.54 | DOWN |
| Dhcr24 | -1.30 | -1.76 | -1.56 | DOWN |
| Rassf8 | -1.29 | -1.98 | -1.94 | DOWN |
| Atp1a2 | -1.28 | -1.31 | -1.24 | DOWN |
| Gja3 | -1.28 | -1.94 | -2.59 | DOWN |
| B3galt1 | -1.28 | -2.49 | -1.86 | DOWN |
| Wnk2 | -1.28 | -2.40 | -1.59 | DOWN |
| Tab2 | -1.27 | -2.44 | -2.09 | DOWN |
| Aaed1 | -1.26 | -1.69 | -1.34 | DOWN |
| St3gal2 | -1.25 | -1.63 | -1.51 | DOWN |
| Gfra4 | -1.25 | -1.14 | -1.35 | DOWN |
| Cuedc1 | -1.25 | -1.91 | -1.63 | DOWN |
| Rabgef1 | -1.25 | -1.76 | -1.47 | DOWN |
| Kctd16 | -1.24 | -1.51 | -1.53 | DOWN |
| Mllt3 | -1.24 | -2.66 | -1.62 | DOWN |
| Tmem143 | -1.23 | -1.13 | -1.02 | DOWN |
| Akap2 | -1.23 | -1.51 | -1.22 | DOWN |
| Ppp3r1 | -1.22 | -1.13 | -1.26 | DOWN |
| Nkiras1 | -1.22 | -1.25 | -1.55 | DOWN |
| Clcn4 | -1.21 | -1.79 | -2.02 | DOWN |
| Stc2 | -1.21 | -1.90 | -1.97 | DOWN |
| Ndufa10l1 | -1.20 | -1.31 | -1.67 | DOWN |
| RGD1311756 | -1.20 | -2.20 | -1.97 | DOWN |
| Rragd | -1.20 | -2.07 | -1.74 | DOWN |
| Zfp358 | -1.19 | -2.44 | -1.94 | DOWN |
| Celsr2 | -1.19 | -2.43 | -2.47 | DOWN |
| Tomm40l | -1.19 | -1.17 | -1.27 | DOWN |
| Dynll2 | -1.19 | -1.21 | -1.05 | DOWN |
| Strbp | -1.19 | -1.59 | -1.29 | DOWN |
| Hfe2 | -1.18 | -1.24 | -1.22 | DOWN |
| Rgs5 | -1.18 | -1.91 | -1.24 | DOWN |
| Ugp2 | -1.18 | -1.40 | -1.07 | DOWN |
| Mb21d2 | -1.18 | -1.31 | -1.20 | DOWN |
| Ccdc91 | -1.17 | -1.26 | -1.19 | DOWN |
| Fhl5 | -1.16 | -1.62 | -1.14 | DOWN |
| Sgms1 | -1.15 | -1.74 | -1.43 | DOWN |
| Olr63 | -1.15 | -1.15 | -1.03 | DOWN |
| Arhgef7 | -1.14 | -1.27 | -1.38 | DOWN |
| Six2 | -1.14 | -1.44 | -1.78 | DOWN |
| ST7 | -1.13 | -1.07 | -1.02 | DOWN |
| RGD1308706 | -1.13 | -1.08 | -1.16 | DOWN |
| Ogdh | -1.12 | -1.48 | -1.29 | DOWN |
| Tead1 | -1.12 | -1.99 | -1.73 | DOWN |
| Fut10 | -1.12 | -1.41 | -1.52 | DOWN |
| Fem1a | -1.11 | -1.55 | -1.30 | DOWN |
| Acsl1 | -1.11 | -1.09 | -1.11 | DOWN |
| Smox | -1.11 | -1.31 | -1.02 | DOWN |
| Smyd1 | -1.10 | -1.31 | -1.05 | DOWN |
| Mfn2 | -1.10 | -1.25 | -1.09 | DOWN |
| Xk | -1.10 | -1.48 | -1.22 | DOWN |
| St3gal3 | -1.09 | -1.63 | -1.55 | DOWN |
| Atmin | -1.09 | -1.53 | -1.40 | DOWN |
| Ppm1a | -1.09 | -1.87 | -1.58 | DOWN |
| Trak1 | -1.08 | -1.39 | -1.39 | DOWN |
| Dnajc28 | -1.08 | -1.59 | -1.49 | DOWN |
| Fam220a | -1.08 | -1.42 | -1.23 | DOWN |
| Galns | -1.07 | -1.35 | -1.04 | DOWN |
| Slc25a26 | -1.07 | -1.21 | -1.01 | DOWN |
| Tmem248 | -1.07 | -1.49 | -1.01 | DOWN |
| Acvr1b | -1.07 | -1.79 | -1.59 | DOWN |
| Kif1c | -1.07 | -2.04 | -1.65 | DOWN |
| Kcnh1 | -1.06 | -1.32 | -1.21 | DOWN |
| Smad6 | -1.06 | -1.22 | -1.36 | DOWN |
| Sema4d | -1.06 | -1.39 | -1.68 | DOWN |
| Nck2 | -1.06 | -1.06 | -1.25 | DOWN |
| Tmem196 | -1.05 | -1.29 | -1.39 | DOWN |
| Gfod1 | -1.05 | -1.96 | -1.87 | DOWN |
| Nup210 | -1.05 | -1.49 | -1.69 | DOWN |
| Bnip1 | -1.04 | -1.51 | -1.03 | DOWN |
| Adrbk1 | -1.03 | -1.61 | -1.07 | DOWN |
| Ppard | -1.02 | -1.30 | -1.24 | DOWN |
| Pde7b | -1.02 | -1.64 | -1.11 | DOWN |
| Det1 | -1.01 | -1.97 | -1.74 | DOWN |
| Dirc2 | -1.01 | -1.28 | -1.27 | DOWN |
| Ptp4a2 | -1.00 | -1.06 | -1.13 | DOWN |
| C1qa | 1.00 | 1.68 | 1.69 | UP |
| Rasgrp1 | 1.01 | 1.09 | 1.19 | UP |
| Gbp5 | 1.01 | 2.68 | 1.14 | UP |
| Trim14 | 1.01 | 1.50 | 1.55 | UP |
| Atg16l2 | 1.01 | 1.24 | 1.24 | UP |
| Sfxn1 | 1.02 | 1.39 | 1.65 | UP |
| Id2 | 1.02 | 1.30 | 1.42 | UP |
| MGC112715 | 1.02 | 1.32 | 1.22 | UP |
| Dctd | 1.02 | 1.73 | 1.46 | UP |
| Lat | 1.02 | 1.58 | 1.17 | UP |
| Dse | 1.03 | 1.07 | 1.19 | UP |
| RGD1310110 | 1.03 | 1.36 | 1.30 | UP |
| Gpr171 | 1.03 | 1.99 | 1.47 | UP |
| RGD1559482 | 1.03 | 1.38 | 1.98 | UP |
| Col3a1 | 1.04 | 1.30 | 1.41 | UP |
| Qprt | 1.04 | 1.25 | 1.32 | UP |
| Cfb | 1.04 | 1.25 | 1.03 | UP |
| Mocos | 1.04 | 1.29 | 1.02 | UP |
| Panx1 | 1.05 | 1.74 | 2.55 | UP |
| Dennd1c | 1.05 | 1.67 | 1.06 | UP |
| Thbs4 | 1.05 | 1.74 | 1.92 | UP |
| Lrp1 | 1.05 | 1.18 | 1.20 | UP |
| Rbl1 | 1.05 | 1.49 | 1.12 | UP |
| Tgfb1 | 1.05 | 1.69 | 1.04 | UP |
| Folr2 | 1.06 | 1.54 | 1.87 | UP |
| Mgp | 1.06 | 1.79 | 1.82 | UP |
| Sema4a | 1.06 | 1.60 | 1.34 | UP |
| Cd44 | 1.07 | 1.14 | 1.44 | UP |
| Dok1 | 1.07 | 1.17 | 1.17 | UP |
| C1s | 1.07 | 1.57 | 1.44 | UP |
| Ccdc80 | 1.08 | 1.50 | 2.04 | UP |
| Tlr5 | 1.08 | 1.51 | 1.36 | UP |
| Derl3 | 1.08 | 1.53 | 1.08 | UP |
| Scrn1 | 1.08 | 1.56 | 1.93 | UP |
| Rab38 | 1.09 | 1.45 | 1.46 | UP |
| RT1-S2 | 1.10 | 1.61 | 1.26 | UP |
| Penk | 1.10 | 2.03 | 1.91 | UP |
| Ndrg4 | 1.10 | 1.37 | 1.80 | UP |
| Plcb2 | 1.10 | 1.45 | 1.01 | UP |
| Lrrc16a | 1.10 | 1.76 | 1.67 | UP |
| Styxl1 | 1.11 | 1.49 | 1.78 | UP |
| Msr1 | 1.11 | 1.57 | 2.24 | UP |
| Glb1l | 1.12 | 1.25 | 1.07 | UP |
| Mmp2 | 1.12 | 1.42 | 1.46 | UP |
| Ttc12 | 1.13 | 1.48 | 1.46 | UP |
| Tap1 | 1.13 | 1.45 | 1.03 | UP |
| Tmem173 | 1.14 | 1.54 | 1.34 | UP |
| Hmha1 | 1.14 | 1.55 | 1.24 | UP |
| Mrc2 | 1.14 | 1.51 | 1.44 | UP |
| Lrrn1 | 1.14 | 1.29 | 1.17 | UP |
| Stambpl1 | 1.14 | 1.59 | 1.27 | UP |
| Vash2 | 1.14 | 2.25 | 1.72 | UP |
| C4b | 1.14 | 1.79 | 1.73 | UP |
| Gins3 | 1.14 | 1.44 | 1.15 | UP |
| Itgb7 | 1.15 | 1.58 | 1.38 | UP |
| Lgals3 | 1.15 | 1.34 | 1.60 | UP |
| LOC308990 | 1.15 | 1.51 | 1.37 | UP |
| Flrt2 | 1.15 | 1.18 | 1.12 | UP |
| Sfrp1 | 1.15 | 1.99 | 1.84 | UP |
| Igfbp4 | 1.16 | 1.71 | 1.39 | UP |
| Ror2 | 1.16 | 1.40 | 1.11 | UP |
| Dhx58 | 1.16 | 1.14 | 1.19 | UP |
| Psmb11 | 1.17 | 1.35 | 1.24 | UP |
| Kif13b | 1.17 | 1.56 | 1.60 | UP |
| Rasal1 | 1.17 | 1.84 | 1.18 | UP |
| Unc13b | 1.17 | 1.16 | 1.37 | UP |
| Pmfbp1 | 1.17 | 2.00 | 1.87 | UP |
| Nsg1 | 1.17 | 1.46 | 1.30 | UP |
| Serping1 | 1.18 | 1.65 | 1.56 | UP |
| Psmb9 | 1.19 | 1.46 | 1.09 | UP |
| Tmed3 | 1.19 | 1.58 | 1.51 | UP |
| Arl5c | 1.19 | 1.33 | 2.30 | UP |
| Lpcat2 | 1.19 | 1.14 | 1.35 | UP |
| Tanc2 | 1.19 | 1.21 | 1.13 | UP |
| Pgm3 | 1.19 | 1.41 | 1.07 | UP |
| Ccr1 | 1.19 | 1.78 | 1.72 | UP |
| Cyb5r2 | 1.20 | 1.20 | 1.40 | UP |
| Fkbp10 | 1.20 | 1.38 | 1.23 | UP |
| Fam105a | 1.20 | 1.80 | 1.20 | UP |
| Mdk | 1.20 | 2.18 | 1.89 | UP |
| Cotl1 | 1.20 | 1.58 | 1.44 | UP |
| LOC691995 | 1.20 | 2.07 | 2.35 | UP |
| Alk | 1.21 | 1.01 | 1.32 | UP |
| Carf | 1.21 | 1.80 | 1.34 | UP |
| Arhgap22 | 1.21 | 1.35 | 1.21 | UP |
| Myl12a | 1.22 | 1.22 | 1.18 | UP |
| Cd83 | 1.22 | 1.68 | 1.28 | UP |
| Cxcl16 | 1.22 | 1.63 | 1.36 | UP |
| Il17ra | 1.23 | 1.38 | 1.31 | UP |
| Slc41a2 | 1.23 | 1.29 | 1.06 | UP |
| Dhfr | 1.23 | 1.47 | 1.16 | UP |
| Zfp382 | 1.23 | 1.58 | 1.30 | UP |
| Aig1 | 1.23 | 1.18 | 1.04 | UP |
| Zfp365 | 1.23 | 1.20 | 2.06 | UP |
| Tmbim1 | 1.24 | 1.42 | 1.79 | UP |
| Ncf4 | 1.24 | 1.40 | 1.24 | UP |
| Lrrc4 | 1.24 | 1.54 | 2.09 | UP |
| Acap1 | 1.24 | 1.95 | 1.11 | UP |
| Zfp90 | 1.24 | 1.48 | 1.21 | UP |
| Olfml3 | 1.25 | 1.77 | 1.44 | UP |
| Bid | 1.25 | 1.41 | 1.31 | UP |
| Kif21b | 1.25 | 1.09 | 1.12 | UP |
| Pdpn | 1.25 | 1.64 | 1.37 | UP |
| Slc39a8 | 1.25 | 1.40 | 1.65 | UP |
| Dapp1 | 1.25 | 1.62 | 1.30 | UP |
| Ctss | 1.25 | 1.89 | 1.80 | UP |
| Rgs18 | 1.26 | 1.40 | 1.17 | UP |
| Tmem71 | 1.26 | 1.23 | 1.56 | UP |
| Srgap2 | 1.26 | 1.69 | 1.03 | UP |
| Nap1l2 | 1.26 | 1.13 | 1.10 | UP |
| Gpr183 | 1.27 | 1.25 | 1.16 | UP |
| Lcp1 | 1.27 | 1.21 | 1.15 | UP |
| Apoe | 1.27 | 1.81 | 1.70 | UP |
| Casp3 | 1.27 | 1.46 | 1.17 | UP |
| Nfkb2 | 1.27 | 1.46 | 1.21 | UP |
| Fibin | 1.29 | 1.27 | 1.92 | UP |
| Rasa2 | 1.29 | 1.92 | 1.62 | UP |
| Cd300le | 1.29 | 1.74 | 1.91 | UP |
| Gpr160 | 1.29 | 1.66 | 1.42 | UP |
| LOC499781 | 1.29 | 1.59 | 1.07 | UP |
| Cd46 | 1.30 | 1.28 | 1.19 | UP |
| Slc40a1 | 1.30 | 1.85 | 1.68 | UP |
| Milr1 | 1.30 | 1.44 | 1.57 | UP |
| Slc17a9 | 1.31 | 1.51 | 1.31 | UP |
| Fcgr2b | 1.31 | 2.08 | 1.90 | UP |
| Timp2 | 1.31 | 1.60 | 1.02 | UP |
| Trpm3 | 1.31 | 1.47 | 1.42 | UP |
| Atpif1 | 1.31 | 1.22 | 1.29 | UP |
| Snx20 | 1.32 | 1.69 | 1.45 | UP |
| Ptpn18 | 1.32 | 1.79 | 1.30 | UP |
| Lbp | 1.32 | 1.64 | 1.63 | UP |
| RGD1311558 | 1.32 | 1.78 | 1.60 | UP |
| Dlk1 | 1.33 | 2.17 | 1.62 | UP |
| Cd37 | 1.33 | 1.58 | 1.47 | UP |
| Fam180a | 1.33 | 2.16 | 2.03 | UP |
| Chrne | 1.33 | 1.73 | 1.20 | UP |
| Ptpro | 1.33 | 1.63 | 1.37 | UP |
| Ftl1 | 1.34 | 1.71 | 1.60 | UP |
| Dmd | 1.34 | 1.25 | 1.01 | UP |
| Nrxn1 | 1.35 | 1.62 | 1.63 | UP |
| Mvp | 1.35 | 1.75 | 1.19 | UP |
| Irf5 | 1.35 | 1.45 | 1.26 | UP |
| Tmem106a | 1.35 | 1.25 | 1.18 | UP |
| Pmel | 1.36 | 1.47 | 1.45 | UP |
| Map4k1 | 1.36 | 1.41 | 1.26 | UP |
| Hck | 1.36 | 1.42 | 1.34 | UP |
| Mrc1 | 1.36 | 2.01 | 1.87 | UP |
| C2 | 1.36 | 1.34 | 1.63 | UP |
| Pld4 | 1.36 | 1.60 | 1.34 | UP |
| Prrx2 | 1.36 | 1.65 | 1.45 | UP |
| Dynll1 | 1.36 | 1.99 | 1.43 | UP |
| Pdk3 | 1.37 | 1.58 | 1.05 | UP |
| Tfcp2l1 | 1.37 | 1.06 | 1.01 | UP |
| Cp | 1.37 | 1.32 | 1.42 | UP |
| Tmem138 | 1.37 | 1.56 | 1.05 | UP |
| Smim5 | 1.38 | 1.26 | 1.54 | UP |
| Apobec1 | 1.38 | 2.04 | 1.77 | UP |
| Klrd1 | 1.38 | 2.15 | 1.82 | UP |
| Clcnkb | 1.38 | 1.74 | 1.75 | UP |
| Syk | 1.39 | 1.82 | 1.49 | UP |
| Flrt3 | 1.39 | 1.69 | 1.66 | UP |
| C5ar1 | 1.39 | 1.70 | 1.58 | UP |
| Usp11 | 1.39 | 1.23 | 1.05 | UP |
| Itgb2 | 1.39 | 1.32 | 1.40 | UP |
| Tspo | 1.39 | 2.72 | 2.14 | UP |
| Srgap3 | 1.40 | 1.65 | 1.32 | UP |
| RT1-CE6 | 1.40 | 1.83 | 1.08 | UP |
| Id4 | 1.41 | 1.30 | 1.83 | UP |
| Ltbp2 | 1.42 | 2.35 | 2.31 | UP |
| Fbln1 | 1.42 | 1.96 | 1.89 | UP |
| Slitrk6 | 1.42 | 1.56 | 1.20 | UP |
| Timp1 | 1.42 | 1.78 | 1.66 | UP |
| C1qtnf6 | 1.43 | 2.02 | 1.11 | UP |
| Jak3 | 1.43 | 1.56 | 1.20 | UP |
| Gdf15 | 1.43 | 2.99 | 4.12 | UP |
| Csf1r | 1.44 | 1.77 | 1.51 | UP |
| Hmgn2 | 1.44 | 1.46 | 1.19 | UP |
| Fn1 | 1.44 | 1.15 | 1.39 | UP |
| Ptgfr | 1.44 | 2.15 | 1.92 | UP |
| Cks2 | 1.44 | 2.87 | 2.46 | UP |
| Bhlhe22 | 1.45 | 2.64 | 2.58 | UP |
| Slfn13 | 1.45 | 1.50 | 1.73 | UP |
| Slc17a5 | 1.45 | 1.01 | 1.17 | UP |
| Mnda | 1.45 | 1.72 | 1.80 | UP |
| Ifitm1 | 1.46 | 1.95 | 1.85 | UP |
| Pcyox1l | 1.46 | 1.51 | 1.23 | UP |
| Csf2ra | 1.46 | 1.33 | 1.32 | UP |
| Renbp | 1.47 | 1.76 | 1.13 | UP |
| Bnc2 | 1.48 | 1.36 | 1.14 | UP |
| Mex3b | 1.48 | 1.36 | 1.24 | UP |
| Iah1 | 1.48 | 1.98 | 1.32 | UP |
| Btk | 1.48 | 1.85 | 1.64 | UP |
| RT1-CE1 | 1.49 | 1.72 | 1.57 | UP |
| Gnb3 | 1.49 | 1.78 | 1.29 | UP |
| Coro1a | 1.49 | 1.72 | 1.32 | UP |
| Fam55b | 1.50 | 1.76 | 1.68 | UP |
| Ckap4 | 1.50 | 1.26 | 1.38 | UP |
| Atp6v1c2 | 1.50 | 2.88 | 1.49 | UP |
| Clec2g | 1.50 | 1.71 | 1.34 | UP |
| Tnfaip8l2 | 1.50 | 1.93 | 1.58 | UP |
| Il17re | 1.50 | 1.77 | 1.78 | UP |
| Dync2h1 | 1.50 | 1.60 | 1.02 | UP |
| Scara3 | 1.51 | 1.35 | 1.40 | UP |
| Figf | 1.51 | 2.16 | 1.45 | UP |
| Col8a1 | 1.51 | 1.84 | 1.90 | UP |
| P2ry6 | 1.51 | 1.69 | 1.26 | UP |
| Tyrobp | 1.52 | 1.90 | 1.75 | UP |
| Serpina3n | 1.52 | 1.84 | 2.17 | UP |
| B9d2 | 1.52 | 1.72 | 1.54 | UP |
| Erbb3 | 1.53 | 1.38 | 1.42 | UP |
| Slc44a1 | 1.53 | 1.46 | 1.19 | UP |
| Itga11 | 1.53 | 1.41 | 1.70 | UP |
| Aebp1 | 1.53 | 1.64 | 1.42 | UP |
| Lilrb3 | 1.54 | 1.90 | 1.99 | UP |
| Cd74 | 1.54 | 1.86 | 1.59 | UP |
| C1qtnf5 | 1.54 | 1.85 | 1.40 | UP |
| Col23a1 | 1.55 | 2.08 | 1.76 | UP |
| Vof16 | 1.55 | 2.04 | 1.46 | UP |
| Dpysl3 | 1.55 | 1.53 | 2.07 | UP |
| Bco2 | 1.55 | 1.52 | 1.09 | UP |
| Car13 | 1.55 | 1.77 | 1.37 | UP |
| Myrf | 1.56 | 1.37 | 1.11 | UP |
| Clec10a | 1.56 | 2.27 | 2.14 | UP |
| Nkg7 | 1.56 | 1.64 | 1.40 | UP |
| P2ry13 | 1.57 | 1.78 | 1.72 | UP |
| Olfml2a | 1.57 | 1.63 | 1.01 | UP |
| Ptprc | 1.57 | 1.86 | 1.55 | UP |
| Trpv3 | 1.57 | 2.51 | 1.47 | UP |
| Arg1 | 1.57 | 2.00 | 1.57 | UP |
| Serpinf1 | 1.57 | 1.83 | 1.66 | UP |
| Laptm5 | 1.58 | 2.01 | 1.45 | UP |
| Mmp19 | 1.58 | 1.59 | 1.20 | UP |
| Lyz2 | 1.58 | 1.78 | 1.75 | UP |
| Psd4 | 1.58 | 1.71 | 1.13 | UP |
| Arhgap9 | 1.58 | 1.75 | 1.29 | UP |
| Lhfpl2 | 1.59 | 1.82 | 1.31 | UP |
| Tec | 1.59 | 1.36 | 1.50 | UP |
| G6pd | 1.59 | 1.22 | 1.12 | UP |
| Sash3 | 1.59 | 2.02 | 1.75 | UP |
| Fuca1 | 1.60 | 1.71 | 1.15 | UP |
| Eci3 | 1.60 | 1.76 | 1.36 | UP |
| RT1-CE14 | 1.60 | 2.54 | 1.41 | UP |
| Cilp2 | 1.60 | 2.55 | 1.73 | UP |
| Cd82 | 1.61 | 1.57 | 1.18 | UP |
| Loxl1 | 1.61 | 2.28 | 2.37 | UP |
| Myo1f | 1.61 | 1.70 | 1.53 | UP |
| Spi1 | 1.61 | 1.61 | 1.18 | UP |
| Cdh2 | 1.61 | 1.45 | 1.16 | UP |
| Trem2 | 1.62 | 1.92 | 1.93 | UP |
| S100a3 | 1.62 | 6.34 | 1.95 | UP |
| Cd4 | 1.62 | 1.76 | 1.46 | UP |
| Lrrc17 | 1.62 | 1.50 | 1.47 | UP |
| Zap70 | 1.63 | 2.21 | 1.61 | UP |
| LOC688765 | 1.63 | 2.00 | 2.07 | UP |
| Gng10 | 1.63 | 1.47 | 1.21 | UP |
| Cdh11 | 1.64 | 1.60 | 1.19 | UP |
| Ciita | 1.64 | 1.58 | 1.18 | UP |
| Acp5 | 1.64 | 1.80 | 1.71 | UP |
| Isg20 | 1.64 | 2.02 | 1.69 | UP |
| Cdkn2a | 1.64 | 3.19 | 2.38 | UP |
| Rac2 | 1.65 | 1.68 | 1.22 | UP |
| Selplg | 1.65 | 1.72 | 1.06 | UP |
| Kif18a | 1.65 | 2.12 | 1.45 | UP |
| Pkp1 | 1.65 | 1.56 | 1.32 | UP |
| Spn | 1.66 | 1.63 | 1.09 | UP |
| Matn4 | 1.66 | 2.95 | 1.95 | UP |
| Igsf6 | 1.67 | 1.52 | 1.65 | UP |
| Fgf7 | 1.68 | 1.28 | 1.55 | UP |
| Hcls1 | 1.68 | 1.73 | 1.15 | UP |
| Ticam2 | 1.68 | 2.06 | 1.70 | UP |
| Sesn3 | 1.69 | 1.42 | 1.79 | UP |
| Nt5dc2 | 1.69 | 1.80 | 1.43 | UP |
| Klhl6 | 1.70 | 1.66 | 1.18 | UP |
| RT1-CE4 | 1.70 | 1.91 | 1.22 | UP |
| RT1-Db1 | 1.71 | 2.04 | 1.76 | UP |
| Clec5a | 1.72 | 2.34 | 2.32 | UP |
| Ppfibp2 | 1.72 | 1.71 | 1.90 | UP |
| Napsa | 1.72 | 1.77 | 1.85 | UP |
| Smim22 | 1.73 | 2.09 | 1.09 | UP |
| Rgs11 | 1.73 | 1.36 | 1.21 | UP |
| Fcer1g | 1.73 | 1.93 | 1.77 | UP |
| LOC687707 | 1.73 | 2.29 | 1.68 | UP |
| Myf5 | 1.74 | 1.75 | 1.13 | UP |
| Klra1 | 1.74 | 2.10 | 2.06 | UP |
| Calml3 | 1.75 | 3.38 | 2.32 | UP |
| Fbxo32 | 1.75 | -1.52 | -1.53 | UP |
| Slc9a9 | 1.75 | 2.00 | 1.57 | UP |
| Tp63 | 1.75 | 1.98 | 1.98 | UP |
| Plxna3 | 1.76 | 1.97 | 1.07 | UP |
| Tfec | 1.76 | 2.24 | 1.98 | UP |
| Nlrp3 | 1.76 | 1.71 | 1.39 | UP |
| Klf5 | 1.76 | 2.27 | 2.20 | UP |
| Ctsk | 1.76 | 1.85 | 1.80 | UP |
| Aif1 | 1.77 | 2.23 | 2.03 | UP |
| Tmsbl1 | 1.77 | 2.26 | 2.35 | UP |
| Sla | 1.77 | 1.69 | 1.36 | UP |
| Cd86 | 1.78 | 2.18 | 2.18 | UP |
| Clec11a | 1.78 | 2.35 | 1.70 | UP |
| Myh3 | 1.78 | 3.77 | 3.66 | UP |
| Lum | 1.78 | 1.99 | 1.95 | UP |
| Cntn2 | 1.78 | 1.55 | 1.54 | UP |
| Arhgap30 | 1.78 | 1.90 | 1.51 | UP |
| Notch2 | 1.78 | 1.97 | 1.55 | UP |
| Gsta1 | 1.79 | 1.37 | 2.12 | UP |
| Mmp14 | 1.79 | 1.37 | 1.14 | UP |
| Sphkap | 1.79 | 1.75 | 2.09 | UP |
| LOC500475 | 1.79 | 1.68 | 1.11 | UP |
| Mboat2 | 1.80 | 1.76 | 1.39 | UP |
| Cilp | 1.80 | 3.12 | 3.43 | UP |
| Prkcb | 1.80 | 2.25 | 1.93 | UP |
| Hsf2bp | 1.81 | 1.87 | 2.33 | UP |
| Ppic | 1.81 | 2.02 | 1.99 | UP |
| Csf2rb | 1.81 | 1.72 | 1.51 | UP |
| Pole2 | 1.81 | 2.05 | 1.86 | UP |
| Slco4a1 | 1.82 | 3.75 | 1.59 | UP |
| Tac3 | 1.82 | 2.56 | 1.21 | UP |
| Cacnb4 | 1.82 | 2.06 | 1.52 | UP |
| Cryl1 | 1.82 | 1.10 | 1.16 | UP |
| Nckap1l | 1.82 | 2.05 | 1.71 | UP |
| Nox4 | 1.83 | 2.36 | 2.50 | UP |
| Tlcd1 | 1.83 | 1.89 | 1.31 | UP |
| Lamc2 | 1.84 | 1.41 | 1.49 | UP |
| Cyth4 | 1.84 | 2.01 | 1.34 | UP |
| Cd300a | 1.84 | 1.91 | 1.44 | UP |
| Ccne1 | 1.85 | 1.16 | 1.15 | UP |
| Plscr1 | 1.85 | 3.08 | 1.29 | UP |
| LOC363337 | 1.85 | 2.39 | 2.16 | UP |
| Il1b | 1.86 | 1.83 | 1.39 | UP |
| Tmem9 | 1.86 | 2.32 | 1.08 | UP |
| Emr1 | 1.86 | 2.02 | 1.80 | UP |
| Tnmd | 1.86 | 1.58 | 1.54 | UP |
| Itgam | 1.87 | 1.90 | 1.87 | UP |
| Gpx3 | 1.87 | 1.67 | 1.69 | UP |
| Wnt10b | 1.88 | 2.60 | 1.30 | UP |
| RT1-A1 | 1.89 | 2.71 | 1.22 | UP |
| LOC497796 | 1.89 | 2.02 | 2.16 | UP |
| Mill1 | 1.89 | 3.15 | 2.88 | UP |
| RT1-Da | 1.90 | 2.12 | 1.78 | UP |
| Zfp57 | 1.91 | 1.41 | 1.55 | UP |
| Cyp2e1 | 1.91 | 1.49 | 1.69 | UP |
| Fgd2 | 1.92 | 2.04 | 1.53 | UP |
| Abca1 | 1.92 | 2.16 | 1.16 | UP |
| Selp | 1.92 | 2.15 | 1.59 | UP |
| Cnp | 1.92 | 1.63 | 1.37 | UP |
| Camk1d | 1.93 | 1.18 | 1.59 | UP |
| Tp53inp1 | 1.93 | 1.97 | 1.90 | UP |
| Impdh1 | 1.93 | 1.74 | 1.09 | UP |
| Crabp2 | 1.94 | 2.15 | 1.93 | UP |
| Slc35e3 | 1.94 | 1.32 | 1.28 | UP |
| Pde7a | 1.94 | 1.84 | 1.32 | UP |
| S100a4 | 1.94 | 3.57 | 2.56 | UP |
| Fam26f | 1.94 | 2.53 | 2.06 | UP |
| Tlr2 | 1.94 | 1.93 | 1.29 | UP |
| Nudt5 | 1.95 | 1.65 | 1.11 | UP |
| St5 | 1.95 | 2.05 | 1.30 | UP |
| Lppr3 | 1.95 | 1.65 | 2.05 | UP |
| RGD1305939 | 1.95 | 2.07 | 2.01 | UP |
| Kcnj9 | 1.96 | 2.09 | 1.89 | UP |
| Evi2b | 1.96 | 2.03 | 1.08 | UP |
| RT1-Ba | 1.96 | 2.05 | 1.54 | UP |
| Bin2 | 1.96 | 2.08 | 1.68 | UP |
| Lck | 1.97 | 2.14 | 1.17 | UP |
| Sdcbp2 | 1.97 | 2.51 | 1.93 | UP |
| Kcnd1 | 1.97 | 1.38 | 1.27 | UP |
| Tnnt2 | 1.98 | 3.79 | 3.23 | UP |
| Ctsw | 1.98 | 3.08 | 2.18 | UP |
| RT1-Db2 | 1.98 | 2.01 | 2.10 | UP |
| Mustn1 | 1.99 | 1.30 | 1.72 | UP |
| Clec4a1 | 1.99 | 1.85 | 1.70 | UP |
| Sdf2l1 | 1.99 | 1.88 | 1.31 | UP |
| Snai2 | 2.00 | 2.03 | 2.00 | UP |
| Col16a1 | 2.00 | 1.94 | 1.78 | UP |
| Htatip2 | 2.00 | 1.32 | 1.02 | UP |
| Ppap2a | 2.00 | 1.66 | 1.10 | UP |
| Fam229b | 2.01 | 1.60 | 1.13 | UP |
| Spon1 | 2.01 | 2.23 | 1.19 | UP |
| Fyb | 2.01 | 1.85 | 1.28 | UP |
| Runx2 | 2.02 | 1.90 | 1.43 | UP |
| Slitrk4 | 2.02 | 2.80 | 2.43 | UP |
| Nfkbie | 2.02 | 3.00 | 1.58 | UP |
| Itk | 2.03 | 2.57 | 1.46 | UP |
| Steap2 | 2.03 | 2.23 | 1.72 | UP |
| Tpd52l1 | 2.03 | 1.13 | 1.22 | UP |
| Neurl3 | 2.03 | 1.80 | 1.31 | UP |
| Gna15 | 2.04 | 2.04 | 1.86 | UP |
| Igf2 | 2.04 | 2.90 | 1.42 | UP |
| Bcat1 | 2.05 | 1.51 | 1.49 | UP |
| Mms22l | 2.05 | 1.91 | 1.46 | UP |
| Cd53 | 2.05 | 2.30 | 2.05 | UP |
| Asns | 2.06 | 1.54 | 1.72 | UP |
| Tlr8 | 2.06 | 1.81 | 2.04 | UP |
| Il21r | 2.06 | 1.86 | 1.06 | UP |
| C4a | 2.07 | 2.42 | 2.16 | UP |
| Cd68 | 2.07 | 2.03 | 1.99 | UP |
| Evi2a | 2.08 | 1.86 | 1.61 | UP |
| Hcst | 2.08 | 3.00 | 2.25 | UP |
| Slmo1 | 2.08 | 2.27 | 1.51 | UP |
| Tifab | 2.09 | 1.84 | 1.43 | UP |
| Lilra5 | 2.09 | 2.45 | 2.52 | UP |
| Cpz | 2.09 | 2.79 | 1.71 | UP |
| Ccl5 | 2.09 | 2.55 | 1.62 | UP |
| Slc11a1 | 2.10 | 1.37 | 1.46 | UP |
| Cd8a | 2.10 | 2.14 | 1.54 | UP |
| Bbc3 | 2.10 | 2.04 | 2.33 | UP |
| Mical1 | 2.11 | 2.60 | 1.34 | UP |
| Gltpd2 | 2.11 | 2.02 | 1.84 | UP |
| Matk | 2.11 | 2.37 | 2.30 | UP |
| Slc25a18 | 2.12 | 2.20 | 1.35 | UP |
| Emr4 | 2.12 | 2.55 | 2.18 | UP |
| Aknad1 | 2.13 | 1.24 | 1.18 | UP |
| Fcgr1a | 2.13 | 2.15 | 1.92 | UP |
| Skap1 | 2.14 | 3.59 | 2.63 | UP |
| Sectm1 | 2.14 | 3.13 | 3.12 | UP |
| RGD1561849 | 2.14 | 1.88 | 2.04 | UP |
| Il10ra | 2.14 | 1.95 | 1.79 | UP |
| Cidea | 2.14 | 2.31 | 1.54 | UP |
| Rcn1 | 2.15 | 1.56 | 1.10 | UP |
| Morn2 | 2.16 | 1.58 | 1.48 | UP |
| RT1-A2 | 2.17 | 2.58 | 1.45 | UP |
| Fut2 | 2.17 | 3.61 | 1.95 | UP |
| B2m | 2.17 | 2.54 | 1.05 | UP |
| Klhl29 | 2.18 | 1.75 | 1.47 | UP |
| Aldh1l2 | 2.18 | 1.83 | 1.55 | UP |
| Klhl10 | 2.18 | 3.39 | 3.16 | UP |
| Rrad | 2.18 | 1.81 | 1.71 | UP |
| RT1-CE10 | 2.19 | 2.46 | 1.51 | UP |
| Mogat1 | 2.19 | 2.32 | 3.70 | UP |
| MGC105567 | 2.20 | 1.70 | 1.66 | UP |
| Hmox1 | 2.20 | 2.02 | 1.28 | UP |
| Tnfrsf14 | 2.21 | 2.57 | 1.42 | UP |
| Orai2 | 2.21 | 1.87 | 1.14 | UP |
| Parm1 | 2.22 | 2.01 | 2.60 | UP |
| Cd7 | 2.22 | 2.87 | 2.67 | UP |
| Ociad2 | 2.23 | 2.26 | 2.38 | UP |
| Sfrp2 | 2.23 | 3.30 | 2.92 | UP |
| Cdh20 | 2.23 | 1.88 | 2.41 | UP |
| Flt3 | 2.24 | 1.94 | 2.03 | UP |
| Apod | 2.24 | 3.47 | 2.68 | UP |
| Srpx2 | 2.24 | 2.37 | 2.08 | UP |
| Bgn | 2.24 | 2.52 | 2.25 | UP |
| Lox | 2.25 | 2.11 | 1.71 | UP |
| Tert | 2.27 | 1.91 | 1.78 | UP |
| Fbxo30 | 2.27 | 2.20 | 1.28 | UP |
| Fgr | 2.27 | 2.47 | 1.33 | UP |
| Pcgf2 | 2.27 | 2.02 | 1.49 | UP |
| Cenpu | 2.28 | 1.78 | 1.95 | UP |
| Gpr132 | 2.28 | 2.15 | 1.61 | UP |
| Fam178b | 2.29 | 1.68 | 1.58 | UP |
| Slc6a12 | 2.29 | 2.20 | 2.43 | UP |
| Gpr114 | 2.29 | 1.89 | 1.24 | UP |
| Pik3r5 | 2.29 | 2.02 | 1.83 | UP |
| Plod2 | 2.31 | 1.83 | 1.25 | UP |
| Tgfb2 | 2.31 | 1.76 | 1.03 | UP |
| Cd22 | 2.32 | 2.88 | 1.78 | UP |
| Slfn3 | 2.33 | 1.02 | 1.74 | UP |
| Ifi27l2b | 2.33 | 1.50 | 1.78 | UP |
| Tspan11 | 2.34 | 2.23 | 1.41 | UP |
| Prr5l | 2.34 | 1.90 | 1.55 | UP |
| RT1-CE2 | 2.34 | 2.63 | 1.74 | UP |
| Tcp11l1 | 2.34 | 1.58 | 1.05 | UP |
| Cd300lf | 2.35 | 2.24 | 1.58 | UP |
| Pamr1 | 2.35 | 1.94 | 2.86 | UP |
| Ptprn | 2.36 | 2.49 | 1.93 | UP |
| Bex1 | 2.37 | 2.48 | 3.08 | UP |
| Cybb | 2.37 | 1.96 | 1.98 | UP |
| Clec12a | 2.37 | 2.43 | 2.06 | UP |
| Nek8 | 2.38 | 2.43 | 1.48 | UP |
| Col1a1 | 2.38 | 1.77 | 1.32 | UP |
| Reep2 | 2.39 | 2.24 | 1.38 | UP |
| Tuba1b | 2.40 | 2.08 | 1.17 | UP |
| Cpxm1 | 2.40 | 2.85 | 2.02 | UP |
| Clec4a3 | 2.41 | 2.27 | 1.95 | UP |
| Wisp2 | 2.41 | 2.81 | 3.27 | UP |
| Necab3 | 2.41 | 4.00 | 1.33 | UP |
| Copz2 | 2.42 | 2.47 | 1.47 | UP |
| Tg | 2.42 | 1.88 | 1.25 | UP |
| Ccl7 | 2.43 | 1.97 | 1.29 | UP |
| Ly86 | 2.44 | 2.16 | 1.51 | UP |
| Slamf8 | 2.44 | 2.37 | 2.29 | UP |
| Chrna5 | 2.45 | 2.17 | 2.61 | UP |
| Ugt1a7c | 2.45 | 2.32 | 2.08 | UP |
| Kcnd3 | 2.45 | 1.96 | 2.23 | UP |
| Galntl5 | 2.45 | 3.23 | 2.87 | UP |
| Oas2 | 2.46 | 1.32 | 2.02 | UP |
| Xcr1 | 2.48 | 2.63 | 2.01 | UP |
| Zmynd15 | 2.48 | 2.27 | 1.90 | UP |
| Scd | 2.48 | 2.71 | 2.03 | UP |
| Klra17 | 2.49 | 2.65 | 2.73 | UP |
| Dpf1 | 2.50 | 2.19 | 2.25 | UP |
| Baiap2l1 | 2.51 | 2.30 | 1.08 | UP |
| Ifnlr1 | 2.51 | 1.99 | 1.75 | UP |
| Mgmt | 2.52 | 3.20 | 3.21 | UP |
| Armcx2 | 2.52 | 3.25 | 1.67 | UP |
| Thy1 | 2.52 | 2.40 | 2.32 | UP |
| LOC100909970 | 2.54 | 3.11 | 1.44 | UP |
| Lst1 | 2.54 | 2.70 | 2.11 | UP |
| Stpg1 | 2.54 | 3.09 | 2.48 | UP |
| Cxcl10 | 2.54 | 2.09 | 1.95 | UP |
| Tlr7 | 2.55 | 2.42 | 2.16 | UP |
| Klrk1 | 2.56 | 1.84 | 1.43 | UP |
| Bcl2a1 | 2.56 | 2.63 | 2.17 | UP |
| Epsti1 | 2.56 | 2.41 | 2.25 | UP |
| Vav1 | 2.58 | 2.49 | 1.77 | UP |
| Me2 | 2.59 | 1.82 | 1.25 | UP |
| Psd | 2.60 | 3.09 | 2.36 | UP |
| Il18 | 2.60 | 2.24 | 1.94 | UP |
| Blk | 2.61 | 1.90 | 1.35 | UP |
| Bcar3 | 2.62 | 1.93 | 1.38 | UP |
| Ninj1 | 2.62 | 1.90 | 1.08 | UP |
| Spats2 | 2.62 | 2.21 | 1.76 | UP |
| Tmem202 | 2.63 | 3.06 | 2.76 | UP |
| Il22ra2 | 2.64 | 6.12 | 2.23 | UP |
| Il1rn | 2.64 | 2.40 | 2.64 | UP |
| Siglec1 | 2.64 | 1.85 | 1.75 | UP |
| Col6a4 | 2.64 | 2.58 | 2.40 | UP |
| Sh3gl3 | 2.65 | 4.25 | 4.10 | UP |
| Lilrb4 | 2.67 | 1.97 | 2.54 | UP |
| Aldh1a7 | 2.67 | 2.36 | 3.11 | UP |
| Acsl4 | 2.72 | 1.67 | 1.02 | UP |
| Igfbp2 | 2.72 | 2.53 | 1.34 | UP |
| Agrp | 2.72 | 2.60 | 2.94 | UP |
| Sema3a | 2.73 | 3.98 | 3.37 | UP |
| Slc26a7 | 2.73 | 3.46 | 2.01 | UP |
| Mybph | 2.73 | 3.90 | 2.02 | UP |
| Lipg | 2.75 | 2.80 | 1.59 | UP |
| Rnf151 | 2.75 | 2.44 | 2.66 | UP |
| Ncr1 | 2.76 | 3.07 | 3.49 | UP |
| Rpl3 | 2.76 | 2.47 | 1.07 | UP |
| Rhbdl2 | 2.77 | 4.10 | 3.92 | UP |
| Vopp1 | 2.77 | 2.24 | 1.56 | UP |
| Gpr34 | 2.77 | 2.65 | 2.17 | UP |
| Clec4a | 2.77 | 2.55 | 3.13 | UP |
| Myo1g | 2.79 | 1.63 | 1.11 | UP |
| Cdc25c | 2.81 | 2.03 | 1.09 | UP |
| Thbs2 | 2.81 | 2.59 | 2.01 | UP |
| Prss39 | 2.81 | 3.69 | 3.44 | UP |
| Ubxn11 | 2.83 | 2.40 | 1.07 | UP |
| Comp | 2.84 | 2.97 | 2.14 | UP |
| Ltbp1 | 2.84 | 2.07 | 1.66 | UP |
| Zpbp2 | 2.84 | 2.26 | 1.53 | UP |
| Tmprss4 | 2.84 | 3.43 | 3.20 | UP |
| Serpinf2 | 2.88 | 1.99 | 1.08 | UP |
| Slc7a11 | 2.88 | 2.66 | 2.51 | UP |
| Lypd6b | 2.88 | 2.10 | 1.81 | UP |
| Nrip3 | 2.89 | 2.13 | 1.29 | UP |
| Sh3bp2 | 2.89 | 2.27 | 1.43 | UP |
| Tubg2 | 2.90 | 2.75 | 1.23 | UP |
| Gstm3 | 2.91 | 2.30 | 1.66 | UP |
| Sit1 | 2.91 | 2.44 | 2.58 | UP |
| Elf3 | 2.93 | 3.09 | 2.99 | UP |
| Eef1a1 | 2.94 | 2.81 | 1.59 | UP |
| LOC365985 | 2.94 | 3.12 | 3.91 | UP |
| Paqr8 | 2.94 | 2.05 | 1.55 | UP |
| LOC100912292 | 2.95 | 3.38 | 1.63 | UP |
| Abcg1 | 2.96 | 2.61 | 1.73 | UP |
| Tmem107 | 2.96 | 2.43 | 1.68 | UP |
| Col11a1 | 2.99 | 2.20 | 1.45 | UP |
| Il18r1 | 2.99 | 2.57 | 3.09 | UP |
| Slamf9 | 3.00 | 2.51 | 2.61 | UP |
| Igsf10 | 3.03 | 2.85 | 2.27 | UP |
| Postn | 3.05 | 2.25 | 1.97 | UP |
| Ctxn3 | 3.05 | 5.97 | 2.99 | UP |
| Kcnip3 | 3.05 | 2.66 | 1.99 | UP |
| Uchl1 | 3.08 | 2.84 | 2.50 | UP |
| Rassf7 | 3.09 | 2.95 | 1.89 | UP |
| Cd84 | 3.10 | 3.11 | 3.21 | UP |
| Myc | 3.10 | 2.61 | 1.53 | UP |
| Cxcr3 | 3.12 | 3.15 | 2.84 | UP |
| Lca5 | 3.12 | 3.45 | 2.82 | UP |
| Gstt1 | 3.12 | 1.45 | 1.22 | UP |
| Tal2 | 3.12 | 2.53 | 1.42 | UP |
| Egfl6 | 3.13 | 2.33 | 2.07 | UP |
| Steap1 | 3.13 | 3.22 | 3.03 | UP |
| Ccr5 | 3.14 | 2.84 | 2.59 | UP |
| Mab21l3 | 3.17 | 2.09 | 2.17 | UP |
| Serpina9 | 3.18 | 5.16 | 4.21 | UP |
| Ydjc | 3.18 | 2.55 | 1.61 | UP |
| Rdh16 | 3.18 | 2.41 | 1.10 | UP |
| C3 | 3.19 | 3.20 | 1.88 | UP |
| Tmem119 | 3.19 | 3.42 | 2.39 | UP |
| C1qtnf3 | 3.19 | 2.60 | 2.46 | UP |
| Cdkn2b | 3.21 | 3.16 | 1.50 | UP |
| Rab31 | 3.21 | 2.32 | 1.69 | UP |
| Nefm | 3.22 | 2.44 | 1.85 | UP |
| Rtkn2 | 3.23 | 4.03 | 3.46 | UP |
| Gpr65 | 3.23 | 3.06 | 4.00 | UP |
| Ephb3 | 3.23 | 4.02 | 1.42 | UP |
| Snx10 | 3.24 | 2.29 | 1.82 | UP |
| Map1a | 3.24 | 2.06 | 1.83 | UP |
| Sox4 | 3.24 | 2.74 | 2.09 | UP |
| Cd180 | 3.26 | 2.63 | 2.32 | UP |
| Lrrc43 | 3.28 | 3.81 | 2.96 | UP |
| Sntn | 3.29 | 3.00 | 2.08 | UP |
| Ankrd55 | 3.29 | 3.95 | 3.90 | UP |
| Galnt6 | 3.30 | 2.40 | 2.34 | UP |
| Tmem200a | 3.31 | 5.07 | 2.75 | UP |
| Sytl1 | 3.39 | 4.19 | 3.07 | UP |
| Mpeg1 | 3.43 | 2.58 | 1.64 | UP |
| Itgal | 3.45 | 1.90 | 1.45 | UP |
| Gpr174 | 3.45 | 4.05 | 3.13 | UP |
| Myl4 | 3.45 | 3.80 | 2.74 | UP |
| Ms4a7 | 3.46 | 4.05 | 3.66 | UP |
| Sectm1b | 3.49 | 2.99 | 2.34 | UP |
| Fam110a | 3.49 | 3.50 | 2.98 | UP |
| Clec7a | 3.51 | 3.46 | 3.29 | UP |
| Anxa8 | 3.51 | 6.10 | 8.14 | UP |
| Fam167a | 3.52 | 2.99 | 2.22 | UP |
| Abcb1b | 3.52 | 3.90 | 5.63 | UP |
| Slpi | 3.52 | 3.24 | 1.34 | UP |
| Acpp | 3.53 | 3.94 | 3.18 | UP |
| Sox2 | 3.55 | 3.43 | 3.41 | UP |
| Rgs1 | 3.55 | 4.24 | 4.58 | UP |
| Mog | 3.55 | 4.22 | 4.22 | UP |
| Erbb2 | 3.56 | 4.22 | 2.62 | UP |
| Cd3g | 3.56 | 3.67 | 3.04 | UP |
| Sh2d2a | 3.58 | 4.04 | 3.80 | UP |
| Spp1 | 3.59 | 3.10 | 1.97 | UP |
| Sh2d4b | 3.59 | 3.16 | 2.44 | UP |
| Slc30a2 | 3.61 | 2.37 | 2.23 | UP |
| MGC105649 | 3.65 | 3.55 | 1.38 | UP |
| Slc22a14 | 3.67 | 6.05 | 5.05 | UP |
| Gstt3 | 3.68 | 2.55 | 2.10 | UP |
| Kcnf1 | 3.68 | 4.55 | 2.69 | UP |
| Scg2 | 3.68 | 3.98 | 3.46 | UP |
| Tp73 | 3.69 | 4.86 | 5.82 | UP |
| RT1-M2 | 3.72 | 4.44 | 3.30 | UP |
| Tph1 | 3.73 | 2.93 | 1.42 | UP |
| Ngfr | 3.73 | 3.22 | 2.51 | UP |
| Rprm | 3.77 | 4.61 | 3.38 | UP |
| Rrh | 3.78 | 4.33 | 4.49 | UP |
| Aif1l | 3.78 | 4.58 | 1.17 | UP |
| Grb7 | 3.89 | 4.28 | 2.33 | UP |
| Cxcl13 | 3.91 | 3.30 | 2.78 | UP |
| Mt2A | 3.92 | 3.46 | 1.70 | UP |
| Pkib | 3.94 | 2.90 | 2.28 | UP |
| Atp1a3 | 3.95 | 3.85 | 4.57 | UP |
| Mdh1b | 3.96 | 4.49 | 3.18 | UP |
| Clhc1 | 3.96 | 4.44 | 4.40 | UP |
| Slc26a2 | 3.97 | 2.85 | 1.25 | UP |
| Chi3l1 | 3.98 | 3.87 | 2.86 | UP |
| Sidt1 | 4.01 | 3.70 | 3.72 | UP |
| Aoah | 4.02 | 3.74 | 3.45 | UP |
| Ccr7 | 4.02 | 3.64 | 2.34 | UP |
| Dnajc6 | 4.05 | 2.80 | 1.55 | UP |
| Cpne9 | 4.05 | 2.76 | 2.57 | UP |
| Hk3 | 4.07 | 3.04 | 2.56 | UP |
| Cd28 | 4.08 | 3.86 | 2.51 | UP |
| Slc17a2 | 4.14 | 3.22 | 3.34 | UP |
| Klra5 | 4.15 | 3.96 | 3.50 | UP |
| Nrk | 4.16 | 4.83 | 4.78 | UP |
| Megf6 | 4.16 | 2.86 | 1.16 | UP |
| LOC654482 | 4.18 | 4.22 | 2.61 | UP |
| Hebp2 | 4.20 | 5.43 | 2.64 | UP |
| Fcrl2 | 4.20 | 4.07 | 2.71 | UP |
| Atg9b | 4.24 | 1.96 | -1.67 | UP |
| Ptprv | 4.24 | 3.94 | 3.14 | UP |
| Chrd | 4.26 | 4.03 | 1.63 | UP |
| Flrt1 | 4.31 | 4.40 | 1.40 | UP |
| Prrt4 | 4.31 | 4.23 | 3.11 | UP |
| Myog | 4.32 | 4.08 | 2.21 | UP |
| Akap14 | 4.33 | 3.46 | 4.12 | UP |
| Tubb6 | 4.35 | 3.03 | 1.98 | UP |
| Rdh7 | 4.41 | 4.32 | 3.13 | UP |
| Ms4a14 | 4.46 | 4.78 | 4.93 | UP |
| Tmem8c | 4.57 | 6.08 | 1.44 | UP |
| P4ha3 | 4.58 | 4.55 | 3.47 | UP |
| Sox11 | 4.59 | 4.01 | 3.09 | UP |
| Faslg | 4.61 | 4.66 | 4.23 | UP |
| Pax1 | 4.62 | 3.85 | 3.80 | UP |
| Adap2 | 4.63 | 4.54 | 1.93 | UP |
| Ibsp | 4.64 | 5.25 | 3.53 | UP |
| Clec2dl1 | 4.67 | 3.34 | 4.57 | UP |
| Sec1 | 4.68 | 4.87 | 1.64 | UP |
| Dio3 | 4.72 | 5.60 | 4.98 | UP |
| Sirpa | 4.74 | 3.82 | 2.35 | UP |
| Pnma1 | 4.82 | 3.31 | 1.27 | UP |
| Prrg4 | 4.83 | 4.34 | 3.74 | UP |
| Ceacam16 | 4.86 | 2.56 | 2.74 | UP |
| Ncam1 | 4.88 | 3.64 | 1.73 | UP |
| Adam19 | 4.93 | 4.44 | 2.18 | UP |
| Abcc6 | 5.03 | 5.71 | 4.01 | UP |
| Slamf6 | 5.05 | 4.82 | 4.58 | UP |
| Slc12a3 | 5.08 | 4.25 | 3.90 | UP |
| Runx1 | 5.12 | 4.22 | 1.71 | UP |
| Cdkn1a | 5.14 | 5.15 | 4.71 | UP |
| Mt1a | 5.17 | 4.91 | 2.30 | UP |
| Artn | 5.19 | 2.67 | 3.22 | UP |
| Cst7 | 5.20 | 5.32 | 1.22 | UP |
| Mmp9 | 5.22 | 4.19 | 3.04 | UP |
| Rbm47 | 5.22 | 2.85 | 1.63 | UP |
| Gadd45a | 5.23 | 4.93 | 2.67 | UP |
| Atp6v0d2 | 5.26 | 5.50 | 5.76 | UP |
| Panx2 | 5.27 | 2.68 | 3.17 | UP |
| Gpnmb | 5.29 | 3.05 | 2.35 | UP |
| Cd8b | 5.34 | 4.97 | 4.34 | UP |
| Plekhg4 | 5.34 | 4.71 | 2.53 | UP |
| Cthrc1 | 5.38 | 4.34 | 3.22 | UP |
| Fst | 5.44 | 5.28 | 5.08 | UP |
| Mgst2 | 5.49 | 2.74 | 1.35 | UP |
| Ankrd1 | 5.49 | 5.43 | 5.38 | UP |
| Krt8 | 5.52 | 4.96 | 2.93 | UP |
| Bglap | 5.53 | 6.67 | 4.79 | UP |
| Grem1 | 5.55 | 5.91 | 4.77 | UP |
| Clrn1 | 5.61 | 6.26 | 3.97 | UP |
| Mmp3 | 5.63 | 3.41 | 3.41 | UP |
| Serpina12 | 5.71 | 3.41 | 1.92 | UP |
| Sln | 5.95 | 6.44 | 5.82 | UP |
| Klk1c7 | 5.97 | 5.59 | 4.49 | UP |
| Chrna1 | 5.97 | 5.45 | 1.62 | UP |
| Nsg2 | 6.00 | 6.43 | 2.05 | UP |
| S100a8 | 6.20 | 5.62 | 2.93 | UP |
| Faah | 6.26 | 5.11 | 1.99 | UP |
| Hsd11b2 | 6.26 | 3.25 | 2.06 | UP |
| Slc51a | 6.32 | 6.50 | 2.15 | UP |
| Dclk1 | 6.48 | 7.01 | 2.31 | UP |
| Tmem158 | 6.49 | 5.34 | 4.24 | UP |
| Cd5l | 6.49 | 4.37 | 4.73 | UP |
| Dhrs9 | 6.89 | 2.45 | 2.58 | UP |
| Ppp1r14c | 6.91 | 5.39 | 2.81 | UP |
| Chrng | 7.08 | 7.56 | 2.51 | UP |
| Emb | 7.14 | 4.86 | 2.32 | UP |
| Tac4 | 7.22 | 6.83 | 2.54 | UP |
| Krt18 | 7.30 | 7.80 | 5.87 | UP |
| Scd1 | 7.35 | 7.78 | 3.13 | UP |
| Sohlh2 | 7.49 | 8.55 | 3.01 | UP |
| Lrrc15 | 7.96 | 6.11 | 4.27 | UP |
| Mmp13 | 8.04 | 6.61 | 4.90 | UP |
| Tnn | 8.05 | 6.12 | 3.77 | UP |
| Scn5a | 8.56 | 7.12 | 3.16 | UP |
| Mmp12 | 8.62 | 7.49 | 7.64 | UP |
| Adra1b | 8.69 | 6.16 | 2.31 | UP |
| RGD1566226 | 8.71 | 7.46 | 3.87 | UP |
| Pde6a | 9.21 | 7.57 | 4.79 | UP |
