# Supplementary material for: Identify differential gene expressions in fatty infiltration process in rotator cuff
Source: J Orthop Surg Res. 2019 May 28;14:158. doi: 10.1186/s13018-019-1182-1 (PMC6537194; doi:10.1186/s13018-019-1182-1)
Supplement: Supplementary file 2 — Table S2. Hub-genes identified by CentiScape (degree ≥ 5) among 1089 DEGs. (DOCX 35 kb) [file 13018_2019_1182_MOESM2_ESM.docx]

Table S2 Hub-genes identified by CentiScape (degree≥5) among 1089 DEGs

| Gene symbol | Degree | log_2_Fold Change | MCODE_Score |
| --- | --- | --- | --- |
| Tmprss11d | 180 | -1.41 | 13.95 |
| Ptprc | 82 | 1.55 | 13.95 |
| Itgam | 76 | 1.87 | 13.95 |
| Mmp9 | 61 | 3.04 | 13.95 |
| Tlr2 | 58 | 1.29 | 13.95 |
| Il1b | 49 | 1.39 | 13.95 |
| Il18 | 42 | 1.94 | 13.95 |
| Ccl5 | 41 | 1.62 | 13.95 |
| Cxcl10 | 37 | 1.95 | 13.49 |
| Ccr7 | 28 | 2.34 | 13.00 |
| Ccr5 | 27 | 2.59 | 13.00 |
| Cxcr3 | 28 | 2.84 | 12.68 |
| Spp1 | 44 | 1.97 | 12.63 |
| Ccr1 | 26 | 1.72 | 12.46 |
| Tgfb1 | 57 | 1.04 | 12.45 |
| Cd86 | 62 | 2.18 | 12.27 |
| Hmox1 | 30 | 1.28 | 12.14 |
| Casp3 | 54 | 1.17 | 12.12 |
| Selp | 26 | 1.59 | 11.86 |
| Apoe | 42 | 1.70 | 11.81 |
| Mmp2 | 30 | 1.46 | 11.74 |
| Cd68 | 59 | 1.99 | 11.11 |
| B2m | 38 | 1.05 | 11.00 |
| Prkcq | 33 | -1.02 | 11.00 |
| Cd28 | 25 | 2.51 | 11.00 |
| RT1-A2 | 23 | 1.45 | 11.00 |
| RT1-CE1 | 21 | 1.57 | 11.00 |
| Thy1 | 26 | 2.32 | 10.72 |
| Mmp3 | 18 | 3.41 | 10.72 |
| Ccl7 | 18 | 1.29 | 10.64 |
| Cd83 | 16 | 1.28 | 10.64 |
| Faslg | 30 | 4.23 | 10.53 |
| Ncam1 | 28 | 1.73 | 10.52 |
| Cd3g | 22 | 3.04 | 10.41 |
| Timp1 | 39 | 1.66 | 10.39 |
| Cd8a | 26 | 1.54 | 10.12 |
| Tlr7 | 25 | 2.16 | 10.00 |
| Tec | 24 | 1.50 | 10.00 |
| Cxcl16 | 19 | 1.36 | 10.00 |
| Cxcl13 | 18 | 2.78 | 10.00 |
| Ldlr | 18 | -1.60 | 10.00 |
| C5ar1 | 17 | 1.58 | 10.00 |
| Mchr1 | 16 | -1.72 | 10.00 |
| Penk | 11 | 1.91 | 10.00 |
| P2ry13 | 10 | 1.72 | 10.00 |
| Tas2r130 | 10 |  | 10.00 |
| Cd8b | 26 | 4.34 | 9.46 |
| Col1a1 | 38 | 1.32 | 9.23 |
| Cd44 | 60 | 1.44 | 9.06 |
| Pik3cd | 44 | -1.40 | 9.00 |
| Pik3r5 | 26 | 1.83 | 9.00 |
| Fcgr2b | 25 | 1.90 | 9.00 |
| Fcer1g | 17 | 1.77 | 9.00 |
| Tlr5 | 16 | 1.36 | 9.00 |
| Hck | 75 | 1.34 | 8.97 |
| Fn1 | 19 | 1.39 | 8.95 |
| Csf1r | 39 | 1.51 | 8.90 |
| Bcl2a1 | 18 | 2.17 | 8.84 |
| Timp2 | 16 | 1.02 | 8.84 |
| Esr1 | 35 | -2.24 | 8.67 |
| Cd53 | 24 | 2.05 | 8.59 |
| Mmp14 | 14 | 1.14 | 8.59 |
| Vav1 | 38 | 1.77 | 8.56 |
| Itk | 30 | 1.46 | 8.56 |
| Igf2 | 25 | 1.42 | 8.00 |
| Fcgr1a | 20 | 1.92 | 8.00 |
| Spi1 | 19 | 1.18 | 8.00 |
| Tgfb2 | 19 | 1.03 | 8.00 |
| Cdh2 | 19 | 1.16 | 8.00 |
| Aldoa | 18 | -1.30 | 8.00 |
| Serping1 | 18 | 1.56 | 8.00 |
| Mmp13 | 18 | 4.90 | 8.00 |
| Ctss | 14 | 1.80 | 8.00 |
| Mog | 10 | 4.22 | 8.00 |
| Bgn | 32 | 2.25 | 7.94 |
| Tyrobp | 47 | 1.75 | 7.86 |
| Jak3 | 49 | 1.20 | 7.84 |
| Flt3 | 28 | 2.03 | 7.82 |
| Fgr | 55 | 1.33 | 7.76 |
| Syk | 85 | 1.49 | 7.73 |
| Zap70 | 74 | 1.61 | 7.73 |
| RT1-Db1 | 26 | 1.76 | 7.71 |
| Lgals3 | 15 | 1.60 | 7.64 |
| Lat | 32 | 1.17 | 7.49 |
| Matk | 30 | 2.30 | 7.42 |
| Col3a1 | 24 | 1.41 | 7.27 |
| Coro1a | 21 | 1.32 | 7.00 |
| Tlr8 | 16 | 2.04 | 7.00 |
| Ctsk | 12 | 1.80 | 7.00 |
| Sar1b | 12 | -1.28 | 7.00 |
| Cybb | 31 | 1.98 | 6.95 |
| Laptm5 | 23 | 1.45 | 6.95 |
| Rac2 | 83 | 1.22 | 6.88 |
| Lck | 71 | 1.17 | 6.83 |
| Lcp1 | 19 | 1.15 | 6.81 |
| Ly86 | 15 | 1.51 | 6.81 |
| Hcls1 | 13 | 1.15 | 6.81 |
| Mmp12 | 10 | 7.64 | 6.81 |
| Itgb2 | 64 | 1.40 | 6.74 |
| Cdkn2b | 17 | 1.50 | 6.61 |
| Runx2 | 21 | 1.43 | 6.59 |
| Bglap | 21 | 4.79 | 6.59 |
| RT1-CE4 | 11 | 1.22 | 6.53 |
| RT1-CE10 | 11 | 1.51 | 6.53 |
| RT1-M2 | 11 | 3.30 | 6.53 |
| Btk | 28 | 1.64 | 6.46 |
| RT1-Db2 | 19 | 2.10 | 6.38 |
| Clec4a3 | 13 | 1.95 | 6.38 |
| Cdkn2a | 12 | 2.38 | 6.38 |
| Cd22 | 10 | 1.78 | 6.38 |
| Fgf7 | 18 | 1.55 | 6.24 |
| Klrd1 | 23 | 1.82 | 6.17 |
| Mpp7 | 20 | -1.02 | 6.11 |
| Ppm1a | 19 | -1.58 | 6.11 |
| Pptc7 | 18 | -1.40 | 6.11 |
| Nfkb2 | 23 | 1.21 | 6.10 |
| ENSRNOG00000015290 | 23 |  | 6.00 |
| Pkm | 23 | -1.49 | 6.00 |
| RT1-Da | 22 | 1.78 | 6.00 |
| Kif18a | 18 | 1.45 | 6.00 |
| Gpd2 | 15 | -2.01 | 6.00 |
| RT1-Ba | 14 | 1.54 | 6.00 |
| Pfkm | 14 | -1.33 | 6.00 |
| Hk3 | 14 | 2.56 | 6.00 |
| Dynll1 | 13 | 1.43 | 6.00 |
| Gng10 | 12 | 1.21 | 6.00 |
| Myo1g | 12 | 1.11 | 6.00 |
| Dynll2 | 10 | -1.05 | 6.00 |
| Erbb3 | 26 | 1.42 | 5.98 |
| Spn | 13 | 1.09 | 5.83 |
| Gna15 | 21 | 1.86 | 5.79 |
| Adcy1 | 14 | -3.27 | 5.79 |
| Adcy9 | 12 | -2.49 | 5.79 |
| Nox4 | 11 | 2.50 | 5.79 |
| Trem2 | 10 | 1.93 | 5.79 |
| Emr1 | 10 | 1.80 | 5.79 |
| Blk | 23 | 1.35 | 5.65 |
| Cdc25c | 12 | 1.09 | 5.57 |
| Lox | 14 | 1.71 | 5.56 |
| Smad6 | 22 | -1.36 | 5.50 |
| Fst | 13 | 5.08 | 5.50 |
| Acvr1b | 13 | -1.59 | 5.50 |
| Chrd | 13 | 1.63 | 5.50 |
| Ncr1 | 11 | 3.49 | 5.50 |
| Map4k1 | 10 | 1.26 | 5.50 |
| Itgb7 | 49 | 1.38 | 5.33 |
| Fgf1 | 16 | -1.45 | 5.07 |
| Aldh1l2 | 21 | 1.55 | 5.00 |
| Aldh1a7 | 20 | 3.11 | 5.00 |
| Rps6ka2 | 19 | -1.75 | 5.00 |
| Myo3b | 18 | -1.62 | 5.00 |
| Epas1 | 17 | -1.19 | 5.00 |
| Pfkfb3 | 14 | -2.47 | 5.00 |
| Tubg2 | 14 | 1.23 | 5.00 |
| Cp | 13 | 1.42 | 5.00 |
| Figf | 13 | 1.45 | 5.00 |
| Xcr1 | 12 | 2.01 | 5.00 |
| Hcst | 12 | 2.25 | 5.00 |
| Pfkfb1 | 11 | -1.14 | 5.00 |
| Gpd1 | 11 | -2.35 | 5.00 |
| Lpin1 | 10 | -2.78 | 5.00 |
| Ppap2a | 10 | 1.10 | 5.00 |
| Myc | 80 | 1.53 | 4.99 |
| C1qa | 17 | 1.69 | 4.91 |
| Notch2 | 15 | 1.55 | 4.76 |
| Acsl4 | 12 | 1.02 | 4.76 |
| Acsl1 | 12 | -1.11 | 4.76 |
| Mstn | 12 | -3.50 | 4.76 |
| Clec7a | 11 | 3.29 | 4.76 |
| Erbb2 | 36 | 2.62 | 4.72 |
| Ccnd2 | 16 | -1.22 | 4.69 |
| Gnao1 | 38 | -2.02 | 4.58 |
| Dync2h1 | 10 | 1.02 | 4.52 |
| Aif1 | 29 | 2.03 | 4.46 |
| Acsl6 | 13 | -1.62 | 4.46 |
| Kif5a | 12 | -1.19 | 4.46 |
| Cish | 10 | -3.51 | 4.46 |
| Prkcb | 24 | 1.93 | 4.36 |
| Actn3 | 20 | -1.90 | 4.29 |
| Myh4 | 22 | -2.48 | 4.23 |
| Myh3 | 21 | 3.66 | 4.23 |
| Cdkn1a | 25 | 4.71 | 4.13 |
| Il10ra | 13 | 1.79 | 4.03 |
| Cd4 | 16 | 1.46 | 4.02 |
| Calml3 | 26 | 2.32 | 4.00 |
| ENSRNOG00000016770 | 23 |  | 4.00 |
| Lum | 20 | 1.95 | 4.00 |
| Pde7b | 16 | -1.11 | 4.00 |
| Pde4a | 16 | -1.90 | 4.00 |
| Pde7a | 16 | 1.32 | 4.00 |
| Pygm | 14 | -1.55 | 4.00 |
| Map2k6 | 13 | -1.24 | 4.00 |
| Comp | 12 | 2.14 | 4.00 |
| Myog | 12 | 2.21 | 4.00 |
| Ngfr | 12 | 2.51 | 4.00 |
| Hopx | 11 | -1.22 | 4.00 |
| Lrrn1 | 11 | 1.17 | 4.00 |
| Lipg | 11 | 1.59 | 4.00 |
| Eef1a1 | 11 | 1.59 | 4.00 |
| Adrbk1 | 11 | -1.07 | 4.00 |
| Lrrc4 | 10 | 2.09 | 4.00 |
| Tnnt2 | 10 | 3.23 | 4.00 |
| Gnb3 | 28 | 1.29 | 3.96 |
| Tert | 18 | 1.78 | 3.96 |
| Ephb3 | 15 | 1.42 | 3.89 |
| Tnn | 20 | 3.77 | 3.78 |
| Dok1 | 14 | 1.17 | 3.78 |
| Eci3 | 13 | 1.36 | 3.78 |
| Cryl1 | 13 | 1.16 | 3.78 |
| Tubb6 | 19 | 1.98 | 3.75 |
| Itga11 | 13 | 1.70 | 3.73 |
| Ccbl1 | 12 | -1.08 | 3.73 |
| Gpt2 | 11 | -2.60 | 3.73 |
| Ptpn18 | 17 | 1.30 | 3.45 |
| Nt5c1a | 13 | -2.99 | 3.43 |
| Abca1 | 11 | 1.16 | 3.43 |
| Tuba1b | 21 | 1.17 | 3.33 |
| Tuba8 | 15 | -1.51 | 3.33 |
| Nck2 | 13 | -1.25 | 3.24 |
| Kcnd3 | 18 | 2.23 | 3.14 |
| Myo1f | 12 | 1.53 | 3.14 |
| C4a | 10 | 2.16 | 3.05 |
| Cacna1e | 11 | -1.65 | 3.00 |
| Lrrc17 | 11 | 1.47 | 3.00 |
| Rasgrp3 | 10 | -1.14 | 3.00 |
| Igfals | 10 | -2.46 | 3.00 |
| Ak1 | 18 | -1.12 | 2.78 |
| LOC365985 | 15 | 3.91 | 2.78 |
| Rbl1 | 18 | 1.12 | 2.67 |
| Myl12a | 15 | 1.18 | 2.67 |
| Oas2 | 18 | 2.02 | 2.56 |
| Rrad | 27 | 1.71 | 2.49 |
| Sirpa | 12 | 2.35 | 2.40 |
| Fzd4 | 10 | -1.44 | 2.40 |
| Pik3c2g | 18 | -1.90 | 2.33 |
| Tab2 | 11 | -2.09 | 2.20 |
| Mdh1b | 14 | 3.18 | 2.18 |
| Srms | 12 | -1.22 | 2.14 |
| Ppp3r1 | 10 | -1.26 | 2.14 |
| Pdk2 | 21 | -1.33 | 2.00 |
| Pkn1 | 11 |  | 2.00 |
| C1s | 10 | 1.44 | 2.00 |
| Ak4 | 18 | -2.62 | 1.95 |
| Dmd | 12 | 1.01 | 1.90 |
| Dlat | 12 | -1.03 | 1.82 |
| Atp2b3 | 13 | -1.40 | 1.75 |
| Me2 | 10 | 1.25 | 1.75 |
| Shh | 17 | -3.47 | 1.68 |
| Scd1 | 11 | 3.13 | 1.61 |
| Scn5a | 18 | 3.16 | 1.33 |
| C3 | 12 | 1.88 | 1.31 |
| Pde6a | 15 | 4.79 | 1.20 |
| Dnajc6 | 11 | 1.55 | 1.20 |
| ENSRNOG00000018712 | 10 |  | 1.20 |
| Atp2a1 | 14 | -1.73 | 1.15 |
| Cyp2e1 | 15 | 1.69 | 0.99 |
| Cacna1s | 20 | -1.15 | 0.97 |
| Gucy2g | 13 | -1.01 | 0.86 |
| Pdk3 | 22 | 1.05 | 0.49 |
